# Spondyloenchondrodysplasia: An enigmatic immuno-osseus type I interferonopathy

**DOI:** 10.70962/jhi.20250035

**Published:** 2025-06-04

**Authors:** Callie C.Y. Wong, Tifenn Wauquier, Carolina Uggenti, Colin Stok, Alice Lepelley, Marie-Louise Frémond, Yanick J. Crow

**Affiliations:** 1 MRC Human Genetics Unit, Institute of Genetics and Cancer, University of Edinburgh, Edinburgh, UK; 2 Laboratory of Neurogenetics and Neuroinflammation, Institute Imagine, INSERM UMR1163, Université Paris Cité, Paris, France; 3 Paediatric Haematology-Immunology and Rheumatology Unit, Necker-Enfants Malades Hospital, AP-HP, Paris, France; 4 Reference Centre for Inflammatory Rheumatism, Autoimmune Diseases and Systemic Interferonopathies in Children (RAISE), Paris, France

## Abstract

Spondyloenchondrodysplasia (SPENCD) is a rare immuno-osseus disease due to biallelic mutations in *ACP5*, resulting in a loss of tartrate-resistant acid phosphatase (TRAP) activity and enhanced type I interferon signalling. While TRAP was identified in the 1950s, *ACP5* was cloned in the 1990s, an *Acp5* knockout mouse was reported in 1996, and >3,000 articles are retrievable on PubMed using the terms “tartrate-resistant acid phosphatase” + “TRAP”, the immunopathology of SPENCD remains unclear. Here we describe the clinical phenotype and molecular architecture of SPENCD, review the biology of TRAP, and consider how TRAP deficiency leads to disturbed innate immunity.

## Introduction

Purple acid phosphatases are a family of enzymes distinguished by their characteristic color, low pH optima, and insensitivity to inhibition by tartrate. Type-5 acid phosphatase, also known as tartrate-resistant acid phosphatase (TRAP), is a lysosomal hydrolase expressed in cells of monocytic lineage, with activity against a wide variety of substrates (1). TRAP was identified in human serum and cells >70 years ago, becoming recognized as an important, albeit nonspecific, marker of both bone disease and macrophage activation (2), and a particular cellular feature of hairy cell leukemia (3). However, despite thousands of publications investigating the biology of TRAP, the precise physiological roles of the enzyme remain incompletely defined.

In 1976, Schorr, Legum, and Oshchorn described two brothers with a skeletal dysplasia notable for the presence of flattened vertebrae (platyspondyly) and islands of chondroid tissue within bone (enchondroma), a combination leading them to coin the novel term spondyloenchondrodysplasia (SPENCD) (4). Individuals with the same skeletal features, together with neurological involvement in the form of spasticity and brain calcification (5, 6), and others manifesting a spectrum of autoimmune phenotypes (7, 8), were subsequently reported. In 2011, two groups described SPENCD to be a genetically homogenous disorder due to biallelic loss-of-function (LOF) mutations in *ACP5*, encoding TRAP, drawing attention to enhanced type I interferon (IFN) signalling as a prominent marker of the disease (9, 10). Of historical note, in their paper, Lausch and colleagues (9) made a molecular diagnosis of SPENCD in a 63-year-old male first reported in 1958 as a 10-year-old child with a skeletal dysplasia and systemic lupus erythematosus (SLE) (11). Subsequently, based on earlier work in mouse (12), it was suggested that the immunological features of SPENCD might relate to a failure of mutant TRAP to dephosphorylate osteopontin (OPN) in plasmacytoid dendritic cells (pDCs), leading to persistent Toll-like receptor 9 (TLR9) signalling (13).

Based on our own work and the published literature, here we provide a summary of the clinical phenotype and molecular architecture of SPENCD, discuss what is known about the function of TRAP, and consider how a loss of TRAP activity might result in a disturbance of innate immunity.

## Spencd

### Clinical phenotype

The clinical phenotype of SPENCD variably involves the skeletal, neurological, and immune systems, each of which we consider below. In so doing, we draw heavily on a detailed survey of 26 patients with a genetic diagnosis of SPENCD published by Briggs et al. (14) in 2016, together with a review of the 90 molecularly proven cases of SPENCD that we have been able to identify in the literature (Table 1 and Table S1). In the absence of a comprehensive prospective survey of affected patients within a defined geographical region, such an approach carries a risk of ascertainment bias and imperfect definition of both the frequency of individual disease features and the full spectrum of the clinical phenotype, as well as precluding the derivation of accurate figures on incidence and prevalence. Further, we highlight a relative paucity of information relating to the natural history of the disease beyond childhood, and thus the need for longitudinal clinical and laboratory follow-up studies into adulthood.

In Briggs et al. (14), the age at which features first necessitated medical consultation varied from birth to 15 years. Similarly, in our review of all published cases, only three patients presented after the age of 15 years, out of 77 individuals where this information was recorded (Table 2). Again, reviewing published data, pregnancy and perinatal histories are apparently almost invariably unremarkable, with most patients apparently demonstrating normal size at birth, although short stature can be evident in the first year of life in some cases. In Briggs et al. (14), two patients died prematurely, one in the first year of life secondary to autoimmune thrombocytopenia (AITP), the other at age 30 years with severe arterial hypertension, heart failure, and gastrointestinal bleeding. A further three deaths were recorded in the published cases that we reviewed, at ages 6 years, 9 years, and 7 mo from sudden unexplained respiratory failure during an Evans syndrome flare, *Escherichia coli* sepsis in the context of pancytopenia, and undefined sepsis. These data suggest that except where autoimmune features are uncontrolled, death is a rare clinical outcome. Briggs et al. (14) also drew attention to sometimes marked variability in expression and disease evolution even between siblings.

**Figure 1:**
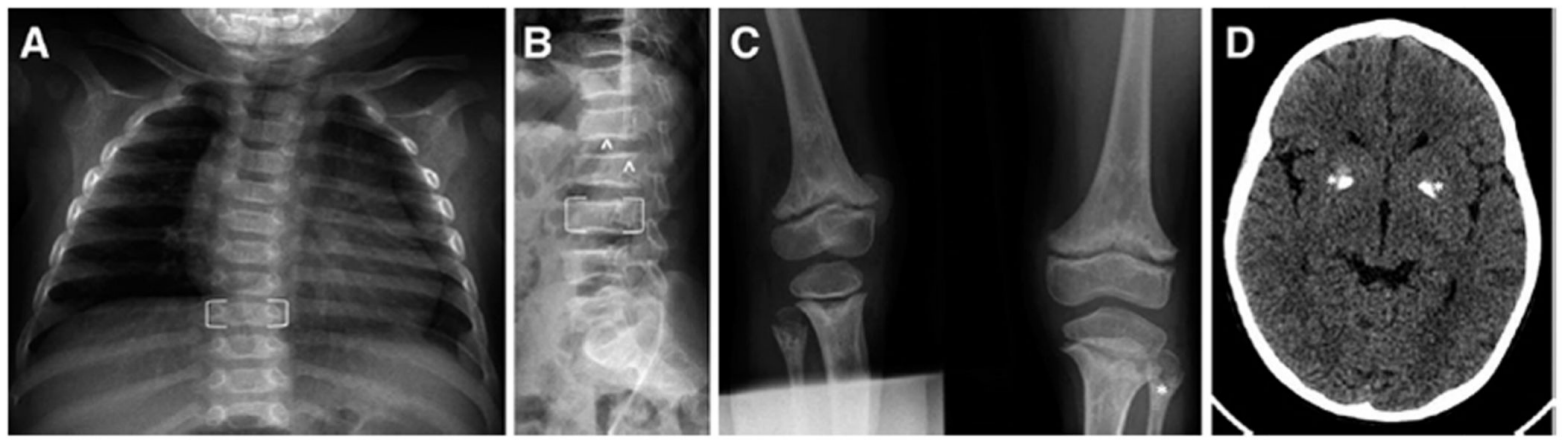
Radiological features of SPENCD. **(A)** Chest X ray showing platyspondyly (representative example indicated by the open brackets) present in a 4-mo-old male homozygous for a c.643G>A (p.(Gly215Arg)) mutation in ACP5. **(B)** Lateral spinal X ray showing platyspondyly (representative example indicated by the open brackets) and irregular vertebral margins (representative example indicated by: ^) in a 10-year-old male homozygous for a c.821T>C (p.(Val247Ala)) mutation in ACP5. **(C)** X ray of femur, tibia, and fibula showing metaphyseal enchondroma (representative example in the left fibula indicated by: *), particularly involving the tibia and fibula of an 11-year-old male homozygous for a c.667C>T (p.(Gln223*)) mutation in ACP5. **(D)** Cranial CT scan showing bilateral basal ganglia calcification (indicated by: *) in the same child as seen in C at the age of 9 years.

The characteristic skeletal radiological features of SPENCD are (1) platyspondyly, with irregularity of both upper and lower end plates and nodular lesions particularly involving the posterior aspect of the vertebral bodies; and (2) radiolucent, ”punched-out” non-ossifying lesions extending from the growth plate into the metaphysis and diaphysis (Fig. 1, A–C). These lesions may be seen in the long bones only (typically, the distal knee, proximal fibula, distal radius, and ulna) or at other sites of endochondral growth (e.g., the iliac Core Research for Evolutional Science and Technology) (15). Individuals with SPENCD may be of reduced stature, due to short trunk and/or limbs depending on the relative degree of spinal and long bone dysplasia. In Briggs et al. (14), skeletal disease, manifest as short stature and/or leg pain/bowing, was the reason for initial presentation in 12 patients (46%), with height varying between the normal range to 6.5 SD below the mean, and an exacerbation of the degree of short stature with age noted in some cases. Importantly, while the presence of characteristic radiological features (even if subtle) significantly increases the likelihood of finding biallelic mutations in *ACP5*, some patients have minimal (e.g., [16]) or no (e.g., [17]) apparent skeletal manifestations (at least in early childhood).

Following the paper by Schorr et al. (4), reports emerged of individuals manifesting both the skeletal features of SPENCD and neurological disease, with spasticity and brain calcification particularly prominent (Fig. 1 D) (5, 6). Briggs et al. (14) observed spasticity in 11 patients (44%), and intracranial calcification on computed tomography imaging in 9 of 13 patients assessed, variably involving the basal ganglia, pons, cerebellar dentate nuclei, and white-grey matter junction. Spasticity can be clinically evident in the first few months of life (e.g., [16, 18]), and intracranial calcification present in early infancy. Briggs et al. (14) also recorded learning difficulties in seven patients (28%). While the latter were typically mild to moderate in severity, marked global impairment (e.g., [19, 20]) and frank neurological regression (20, 21) can occur. In contrast to Aicardi–Goutières syndrome (AGS), significant cerebral white matter disease is apparently unusual. Childhood moyamoya disease has been reported in two siblings with SPENCD and SLE (22).

Immunological disease was the most common reason for first seeking medical attention reported in Briggs et al. (14), being so in 13 patients (50%). Further, at least one autoimmune diagnosis was recorded in 22 patients overall (85%), with 9 (34%) assigned 3 or more such diagnoses. In order of frequency, AITP, SLE, autoimmune hemolytic anemia (AIHA), and hypothyroidism were observed in 12 (46%), 9 (36%), 7 (27%), and 5 (19%) cases, respectively, with other clinical diagnoses including juvenile arthritis, Sjögren syndrome, polymyositis, and coeliac disease. AITP prompted initial presentation in 5 (19%) patients, and among the 12 patients with AITP in the series overall, 7 had a diagnosis of SLE. The high frequency of AITP and AIHA can lead to a diagnosis of Evans syndrome (17, 18, 23). AITP in SPENCD is characteristically refractory to treatment; Briggs et al. (14) recording death in infancy in one patient and cerebral hemorrhage in two further patients. While 9 (34%) patients reported by Briggs et al. (14) fulfilled American College of Rheumatology criteria for a diagnosis of SLE, 21 of the 22 (95%) patients tested in the cohort were positive for the presence of antinuclear antibodies, and 15 of 21 (71%) were anti-dsDNA antibody positive. Likely related to immune dysfunction, four patients experienced severe eczema, two had vasculitis, and one sclerodermatous/acrocyanotic changes leading to digital auto-amputation. Skin biopsy in one patient showed a perivascular polymorphonuclear infiltrate without evidence of deposition of complement or immunoglobulin, consistent with a nonspecific leukocytoclastic vasculitis. Other skin manifestations included Raynaud’s phenomenon and vitiligo. Similarly, high rates of autoimmunity have been observed in molecularly confirmed cases of SPENCD reported in the literature overall (Table S1).

Given the phenotypic overlap with some cases of AGS (particularly, spasticity and intracranial calcification) and the high frequency of SLE (a disorder known to be associated with enhanced type I IFN signalling) in 2011 using genome-wide microarray analysis, Briggs et al. (10) demonstrated increased expression of IFN-stimulated genes (ISGs) in a cohort of molecularly confirmed cases of SPENCD. In a follow-up paper in 2016, these authors extended their results, showing persistent elevation of ISG expression in the whole blood of 9 of 11 patients tested, and persistently increased levels of serum IFN*α* activity in all 10 patients assessed (14). More recently, in unpublished data, we have recorded highly elevated levels of IFN*α* protein in four patients with SPENCD, one of whom manifests no clinical autoimmunity at age 28 years. In the two patients with normal ISG expression ascertained by Briggs et al. (14), one was assessed at the age of 35 years in the absence of any clinical autoimmune disease (his phenotype characterized by typical skeletal features, spasticity, basal ganglia calcification, and moderate intellectual disability). The second patient, with a long history of bone, brain, and immunological disease (including a diagnosis of SLE and lupus nephritis), was on maintenance immunotherapy of prednisone and mycophenolate mophetil when tested, the result being negative on both occasions. Taken as a whole, these findings indicate that enhanced ISG expression is a consistent, albeit not invariable (at least into adulthood), feature of SPENCD, and that ISG expression is likely driven, at least in part, by IFN*α*. As such, SPENCD has been classified as a type I interferonopathy (24).

In 2003, Roifman and Melamed reported four patients with what they considered to be a novel syndrome of combined humoral and cellular immune deficiency, autoimmunity, and spondylometaphyseal dysplasia (25). A further similar case was published in 2007 (26). This cohort of five patients experienced recurrent upper respiratory tract infections, pneumonia, severe varicella infection, disseminated herpes zoster, tuberculosis, Campylobacter enteritis, fatal encephalitis, and recurrent sinusitis. Immune testing revealed variable immunoglobulin levels, with low levels of specific antibodies to tetanus, polio, and pneumococcus following vaccination. Meanwhile, severe varicella infection suggested an immune cellular deficiency, with low total numbers of circulating T lymphocytes and poor ex vivo T cell function. While, to our knowledge, these patients have not been proven molecularly to harbor mutations in *ACP5*, subsequent review of the skeletal findings suggested SPENCD as the likely diagnosis (15). In Briggs et al. (14), significant bacterial and viral infections were recorded in five patients (19%), including recurrent pneumonia, disseminated herpes zoster, skin, and dental abscesses. Although the differentiation of disease-related immunodeficiency from immune defects secondary to treatment can be difficult, low lymphocyte counts (variably involving T, B, and NK cells) and/or hypogammaglobulinemia were recorded in three patients who had not received immunosuppressive therapy (and in nine treated patients).

### Molecular genetics

**Figure 2:**
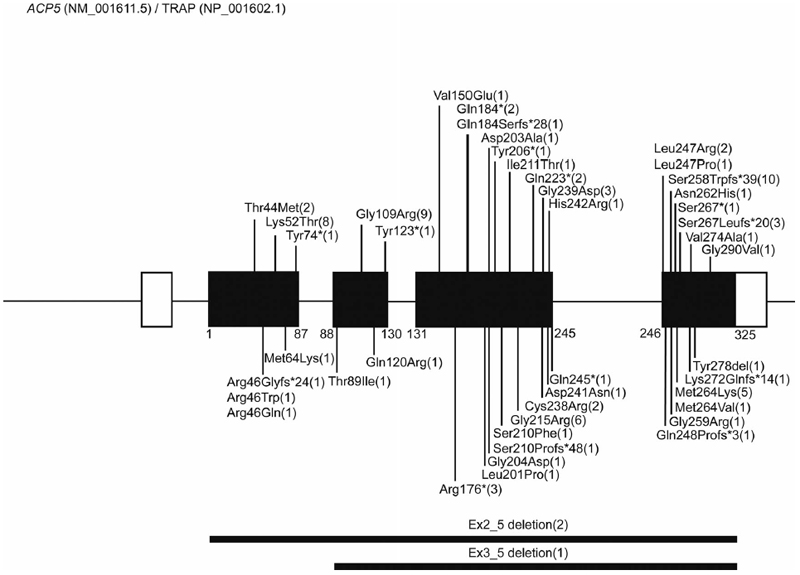
Cartoon of *ACP5* pathogenic mutations reported in patients with SPENCD. Distribution of ACP5 pathogenic variants based on the reports annotated in Table S1. ACP5 (NM_001611.5) comprises five exons, encoding a 325-amino acid protein TRAP (NP_001602.1) translated from exons 2 to 5. Numbers in brackets indicate the number of families in which a mutation has been reported. For the p. Ser258Trpfs*39 mutation, note that seven pedigrees originate from the same small village (28).

In 2011, in back-to-back papers, Lausch et al. (9) and Briggs et al. (10) described SPENCD to result from biallelic mutations in *ACP5*, reporting 14 patients from 11 families and 10 patients from eight families, respectively. In cohorts highly enriched for parental consanguinity, both groups used homozygosity mapping to identify homozygous and compound heterozygous missense substitutions of evolutionarily conserved residues, as well as truncations and deletions predicted to act as null alleles through nonsense-mediated decay of mRNA, loss of protein stability, or loss of functional domains. The observation of biallelic null alleles, including one case homozygous for a complete gene deletion (10), is consistent with the viability of the *Acp5*-null mouse (27). Based on the data presented in 27 publications, we provide an overview of the *ACP5*/TRAP mutational spectrum (Tables S1 and S2; and Fig. 2).

*ACP5* (NM_001611.5) comprises five exons, with the 325-amino acid protein TRAP (NP_001602.1) translated from exons 2 to 5 (29). Four alternative promotors exist within the first three exons, with the distinct expression patterns of the *ACP5* 5′-UTRs suggesting that mRNA expression is regulated using alternative tissue- and cell-dependent promotors (30). The observation of a marked enrichment for consanguinity in reported cases is in keeping with the low minor allele frequency of pathogenic heterozygous variants in control populations. We list 45 distinct mutations (28 missense substitutions; 8 STOP and 7 frameshift mutations; 2 large deletions) distributed throughout the gene (Fig. 2). No genotype–phenotype correlations have been noted. Eight mutations were observed in more than one pedigree, while the remainder were private to individual pedigrees. We are unaware of any patients conforming to the classical SPENCD phenotype being negative for mutations in *ACP5*, suggesting that the disease is genetically homogeneous.

TRAP exists as two isoforms: isoform 5a is a 35-kD monomer, while 5b is a dimer, comprising 23- and 16-kD subunits linked by disulfide bonds, derived by posttranslational proteolytic cleavage of a central regulatory loop domain in isoform 5a (31, 32). Mutations in *ACP5* have been shown to result in negligible levels of total TRAP protein in patient plasma (10) and primary cells (including functionally relevant dendritic cells: DCs) (9). Further, TRAP activity (both total and isoform 5b alone) was shown to be undetectable in serum and leukocyte homogenates from patients, with levels reduced to ~50% in heterozygous parents (9). Ramesh et al. (33) characterized the eight missense substitutions (K52T, T89I, G109R, L201P, G215R, D241N, N282H, and M264K) reported by Lausch et al. (9) and Briggs et al. (10), showing that while expressed, all mutants lacked enzymatic activity. None of the mutant proteins were proteolytically processed into isoform 5b intracellularly, and only three mutants were secreted in significant amounts into the culture medium as the intact 5a isoform. Summarizing, these findings strongly support TRAP LOF as the basis of SPENCD.

## TRAP

### TRAP function

TRAP, a metalloenzyme with a mixed valency di-iron center required for its catalytic activity (34), is expressed primarily by differentiated cells of the mononuclear phagocytic system across a wide variety of tissues in humans and mice (35, 36). The immuno-osseus features of SPENCD are consistent with the expression of TRAP in macrophages, DCs, and osteoclasts. These phagocytic cell types, which originate from myeloid progenitor cells in the bone marrow, breakdown engulfed material through the actions of hydrolytic enzymes and reactive oxygen species (ROS). Indeed, as well as possessing phosphatase activity at an acidic pH optimum, TRAP can generate ROS at neutral pH (37, 38), with mutant phosphatase-dead TRAP enzyme still able to generate ROS (39). Considering the neurological features of SPENCD, while TRAP protein has been reported to be expressed in rat neurons (40), *ACP5* is only minimally expressed in human neurons and microglia (https://brainrnaseq.org/). TRAP 5a, a less active enzyme compared with 5b, is secreted by macrophages and DCs, with isoform 5b remaining intracellular. As such, serum TRAP5a serves as a marker of systemic inflammation (41, 42). In contrast, osteoclasts secrete TRAP5b (43), where cleavage of isoform 5a to generate the 5b dimer can be mediated by cathepsin K (44).

*Acp5*-null mice are viable under laboratory conditions but suffer from developmental deformities of the limb and axial skeleton (27). Reminiscent of the skeletal features seen in patients with SPENCD, these defects include disorganized growth plates indicative of a role for TRAP in endochondral ossification, and mild osteopetrosis due to a resorptive defect of osteoclasts resulting in defective bone remodelling. The bones of *Acp5*-null mice are stronger than those of wild-type animals (45), consistent with the absence of an increased risk of fractures in SPENCD. In contrast, mice overexpressing TRAP develop mild osteoporosis (46). TRAP is found in transcytotic vesicles in cultured osteoclasts (38), and osteoclasts from *Acp5*-null mice demonstrate an accumulation of cytoplasmic vesicles, suggesting a role for TRAP in intracellular vesicular homeostasis (47).

In humans, activated alveolar macrophages (48, 49), monocytes transformed to macrophages by culture in serum-supplemented medium (29) and blood-derived DCs matured by induction with LPS (36) all express abundant TRAP activity. TRAP-deficient macrophages from *Acp5*-null mice demonstrate an altered cytokine profile and a decreased ability to clear bacteria after infection (50, 51). While TRAP has no obvious intrinsic function in T and B cell development, compared with control cells, *Acp5*-null DCs express less MHC class II and CD80, and more IL-10, following LPS stimulation. Th1 responses are also impaired in *Acp5*-null mice, suggesting that TRAP regulates DC maturation and thus naïve T cell responses to DC-presented antigens (52).

### Immunopathology of TRAP deficiency

The data presented in the previous section are consistent with a role of TRAP in immunity, possibly most relevant to the infectious susceptibility observed in some patients with SPENCD. Notably, *Acp5*-null mice have not been described to manifest overt autoimmunity, which is such a prominent feature of *ACP5*-related disease in humans. Neither has neurological involvement been documented in these mice, with the spasticity and intracranial calcification seen in SPENCD typical of a subset of type I interferonopathies (53). Thus, the question remains as to how a loss of TRAP activity leads to autoimmunity and enhanced type I IFN signalling.

Currently, to our knowledge, the only data directly addressing a link between TRAP deficiency and type I IFN signalling were published by An et al. (13) in 2017. Based on earlier work suggesting that intracellular OPN couples the TLR9/MyD88 signalling complex to IFN*α* expression in pDCs in mice (12), An et al. (13) suggested that the autoimmune features of SPENCD might relate to a failure of mutant TRAP to dephosphorylate OPN in pDCs in humans, leading to persistent TLR9 signalling. Perhaps the best-defined substrate of TRAP, OPN (54), known also as T lymphocyte activation-1 (Eta-1), is a phosphorylated acidic glycoprotein secreted in many body fluids and expressed by a variety of cell types, including osteoclasts and activated T cells (55). Dephosphorylation of OPN by TRAP is critical for osteoclast migration (56, 57) and also plays a role in cell-mediated immunity against intracellular viruses and bacteria through the regulation of phosphorylation-dependent interactions of OPN with integrin β3 and CD44 (58). Using confocal microscopy, An et al. (13) observed Golgi localization of OPN and TRAP and partial co-localization of antibody-stained endogenous TRAP and OPN in human primary monocyte-derived macrophages and in the Gen 2.2 model of human pDCs (59). Yeast 2-hybrid screening using a human macrophage cDNA library suggested an interaction of TRAP with OPN, confirmed by reciprocal coprecipitation of OPN and TRAP upon over-expression in HEK293 cells and endogenously expressed in lysates of monocyte-derived macrophages. These authors went on to show that recombinant human OPN was dephosphorylated by recombinant human TRAP at three phosphoserine residues. Further, CpG-A stimulation in a pDC line with stable knockdown of TRAP resulted in increased expression of IFN*α* protein and ISGs and increased IRF7 nuclear translocation, while CpG-B stimulation led to the enhanced production of TNF and IL6, and NF-kB nuclear translocation. In TRAP-knockdown THP-1 cells treated with PMA to generate macrophage-like cells, these authors also observed elevated levels of hyperphosphorylated OPN compared with controls, involving two of the three phosphoserines identified in their experiments with recombinant OPN and TRAP.

While the above findings are of interest, An et al. (13) were unable to directly assess OPN phosphorylation in Gen 2.2 cells due to limited substrate availability in this pDC line. As such, it remains to be formally determined if OPN is a physical component of the TLR9/MyD88 complex in these cells and/or whether TRAP acts on a different substrate(s) in this or other pathway(s). For example, given that TRAP is a lysosomal enzyme it might play a role in TLR7 and TLR9 signalling in pDCs (60, 61), or in STING degradation in macrophages and other immune cells (62, 63). In terms of phenotype, the degree of autoimmunity seen in SPENCD is more reminiscent of gain-of-function mutations in *UNC93B1 and TLR7* (reviewed in [60]), where severe neurological involvement can also be seen (64, 65), rather than disease due to defective processing or enhanced sensing of cytosolic nucleic acid (as in AGS or STING gain-of-function), where frank autoimmunity is less prominent. Although any relevance to SPENCD is currently unknown, it is of possible note that both DNA and RNA oligonucleotides induce TRAP expression in human osteoclasts and monocytes independent of TLR9 (66), that lysosomal proteases can play a role in antigen processing (67), and that both ATP and ADP are TRAP substrates (68, 69).

Another possible mechanism linking TRAP to type I IFN signalling might relate to its function in the removal of mannose-6-phosphate (M6P) from acid hydrolases, enzymes that catabolize macromolecules, extracellular material, and pathogenic organisms within the lysosome. M6P is a posttranslational modification critical to the activation of >60 acid hydrolases, which traffic in clathrin-coated vesicles from the Golgi to the lysosome. Together with lysosomal acid phosphatase (encoded by *ACP2*), TRAP (which is itself transported in a M6P-dependent manner) removes M6P in the lysosome, with an accumulation of M6P-marked proteins observed in *Acp5*-deficient mice (54, 70, 71, 72). Two such M6P-modified glycoproteins are DNase II and ribonuclease T2 (73). Notably, loss of activity of these enzymes is each associated with a type I interferonopathy disease state (74, 75). M6P retention may impair the trafficking and distribution of these enzymes within cells, although the effect on their activity is unknown. Finally, ADA2, encoded by *CECR1*, is another lysosomal enzyme (76), deficiency of which can result in an upregulation of type I IFN signalling (77, 78), possibly through a disturbance of the TLR9 axis (79).

## Concluding remarks

Given the very high frequency of severe autoimmune phenotypes in SPENCD and the possible enrichment of *ACP5* variants in cohorts of patients with SLE (13, 80), understanding the pathogenesis of SPENCD is of high interest. Data suggest a role for TRAP in regulating a putative OPN/TLR9/MyD88 signalosome in pDCs, while we note here that TRAP is also expressed in B cells (https://www.proteinatlas.org/ENSG00000102575-ACP5/single+cell). Type I IFN signalling is enhanced in the majority of patients tested, although whether this is a driver of pathology or simply represents a disease biomarker remains unclear. The apparent efficacy of JAK1/2 /JAK1/3 inhibition in affected patients (Table S3), particularly relating to features of systemic autoimmunity (e.g., [18, 23, 81]) if not neurological involvement (20), is possibly instructive in this regard. Clearly, future studies aimed at better defining the immunopathology of SPENCD are warranted, both in terms of our understanding of fundamental immunological principles and the development of targeted approaches to the treatment of this enigmatic immuno-osseous dysplasia.

## Online supplemental material

Table S1 shows the summarized demographic, genetic, and clinical data available for 90 molecularly proven cases of SPENCD described in 27 reports in the literature. Table S2 shows the information on individual mutations recorded in the 27 references noted in Table S1. Table S3 shows the data provided on patients recorded in Table S1 treated using JAK inhibition.

## Supplementary Material

Supplementary

## Figures and Tables

**Table 1:** Frequency of clinical and laboratory features in patients with SPENCD

Clinical feature	Number of patients^a^	Percentage as reported in Briggs et al. (14)
Skeletal dysplasia	85	96% (24/25)
Short stature	73	96% (24/25)
Brain calcification	36	64% (9/14)
Developmental delay	29	28% (7/25)
SLE	27	36% (9/25)
Spastic paraparesis	25	44% (11/25)
AIHA^b^	22	28% (7/25)
AITP^b^	18	46% (12/26)
Hypothyroidism	14	20% (5/25)
“Significant” infections	11	20% (5/25)
Laboratory feature		
Antinuclear antibody positive	33	95% (21/22)
Anti-double-stranded DNA antibody positive	21	71% (15/21)

a Data from the 90 patients reported in the 27 references included in Table S1. Note that data on each feature was not provided for all 90 patients described in these 27 references.

b A diagnosis of Evans syndrome was made in five patients.

**Table 2:** Age at presentation recorded in patients in the 27 references noted in Table S1 ^a^

Range of age at presentation	Number of patients
<1 year old	17
1–5 years old	32
5–15 years old	25
>15 years old	3

a Note that age at presentation was not provided for all 90 patients.
