# Supplementary material for: Spondyloenchondrodysplasia: An enigmatic immuno-osseus type I interferonopathy
Source: J Hum Immun. 2025 Jun 4;1(2):e20250035. doi: 10.70962/jhi.20250035 (PMC7618195; doi:10.70962/jhi.20250035)
Supplement: Table S1 — shows the summarized demographic, genetic and clinical data available for 90 molecularly proven cases of SPENCD described in 27 reports in the literature. [file jhi_20250035_tables1.docx]

**Supplementary Table 1. Summarised demographic, genetic and clinical data available for 90 molecularly proven cases of SPENCD described in 27 reports in the literature***

| **PMID** | **Patient number** | **Sex** | **Age at onset (years)** | **Siblings** | **Mutation(s)** | | **Phenotype** |
| --- | --- | --- | --- | --- | --- | --- | --- |
|  |  |  |  |  | Nucleotide | Amino acid |  |
| Lausch et al. (1) | P1 | M | 9.5 | No | c.325G>A/c.831_833delCTA | p.Gly109Arg/p.Tyr278del | SS, SD, SLE, APL, steroid-dependent neutropenia, thrombocytopenia, no neurologic signs, normal mental development |
|  | P2 | NA | 1 | Yes (P3) | c.800delC (hom) | p.Ser267* (hom) | SS, SD, frequent fevers, granulocytopenia, ANA, SP, mild DD |
|  | P3 | NA | 12 | Yes (P2) | c.800delC (hom) | p.Ser267* (hom) | SS, SD, granulocytopenia, ANA, no neurologic signs, normal development |
|  | P4 | NA | 3 | No | c.325G>A/c.602T>C | p.Gly109Arg/p.Leu201Pro | Borderline SS, SD, thrombocytopenia, leukopenia, HSM, ANA, BC, SP, normal development |
|  | P5 | NA | 12 | No | c.791T>A (hom) | p.Met264Lys (hom) | SS, SD, frequent fevers, HSM, SLE, ANAs+, no neurologic signs, normal development |
|  | P6 | NA | 4 | No | c.222C>A (hom) | p.Tyr74* (hom) | Mild SS, SD, chronic thrombocytopenia, no neurologic signs, normal development |
|  | P7 | NA | 4 | No | c.784A>C (hom) | p.Asn262His (hom) | Marked SS, SD, AIHA, optic neuritis, ataxia, hemiplegia attributed to vascular infarction or multiple sclerosis, BC, DD |
|  | P8 | F | 16 | Yes (P9) | c.325G>A (hom) | p.Gly109Arg (hom) | SS, SD, SLE with class V lupus nephritis, arthralgia, vitiligo, no neurological signs, BC, mild DD |
|  | P9 | M | 5 | Yes (P8) | c.325G>A (hom) | p.Gly109Arg (hom) | SS, SD, arthritis, ANA, SLE with class IV lupus nephritis, no neurological signs, BC |
|  | P10 | NA | 7 | No | c.643G>A (hom) | p.Gly215Arg (hom) | SS, SD, no extra-skeletal signs, no neurological signs, BC |
|  | P11 | NA | 14 | No | c.643G>A (hom) | p.Gly215Arg (hom) | Moderate SS, SD, chronic thrombocytopenia, ataxia, BC |
|  | P12 | NA | 0.83 | No | c.155A>C (hom) | p.Lys52Thr (hom) | Marked SS, SD, recurrent fever, Kawasaki-like disease, thrombocytopenia and haemolytic anaemia, SLE, ANA, vitiligo, hypothyroidism, SP, seizures, microcephaly, DD |
|  | P13 | NA | 12 | Yes (P14) | c.618C>A (hom) | p.Tyr206* (hom) | Marked SS, SD, rheumatic fever with Sydenham’s chorea |
|  | P14 | NA | 13 | Yes (P13) | c.618C>A (hom) | p.Tyr206* (hom) | SS, SD |
| Briggs et al. (2) | P15 | F | 3 | No | 11,544,822–11,556,767 del (hom) | p.Ex2_5 del (hom) | SD, hypothyroidism, Raynaud's/vasculitis, Sjögren's syndrome, inflammatory myositis, seizures, ANA, deceased at age 30 years due to GI bleeding, heart failure and severe hypertension |
|  | P16 | F | 1 | Yes (P17) | c.369C>A/c.721G>A | p.Tyr123*/p.Asp241Asn | SD, thrombocytopenia, SLE with lupus nephritis, hypothyroidism, DD, ANAs, anti-dsDNA |
|  | P17 | M | 3 | Yes (P16) | c.369C>A/c.721G>A | p.Tyr123*/p.Asp241Asn | SD, AITP |
|  | P18 | M | 2 | Yes (P19) | c. 266C>T (hom) | p.Thr89Ile (hom) | SD, Raynaud's/vasculitis, SP, vasculitic skin rash, ANA, anti-dsDNA |
|  | P19 | F | 14 | Yes (P18) | c. 266C>T (hom) | p.Thr89Ile (hom) | SS, SD, Raynaud's/vasculitis |
|  | P20 | M | 0.67 | No | c.667C>T (hom) | p.Gln223* (hom) | SS, SD, AIHA, ANAs, anti-dsDNA |
|  | P21 | M | 2 | No | 11,543,690–11,548,656 del (hom) | p.Ex3_5 del (hom) | SD, recurrent infections (including pneumonia), thrombocytopenia, SLE with lupus nephritis, ANAs, anti-dsDNA |
|  | P22 | F | 3 | No | c.791 T>A (hom) | p.Met64Lys (hom) | SD, recurrent infections (including pneumonia), SLE, hypothyroidism, thrombocytopenia, non-erosive arthropathy, ANA, anti-dsDNA |
|  | P23 | F | 6 | No | c.643 G>A (hom) | p.Gly215Arg (hom) | SD, thrombocytopenia, AIHA, SLE with lupus nephritis, ANAs, anti-dsDNA |
|  | P24 | F | 4 | No | c.772-790del (hom) | p.Ser258Trpfs*39 (hom) | SD, leg pain, ANAs, anti-dsDNA |
| Girschick et al. (3) | P25 | F | 0.5 | No | c.131C>T/c.816dupC | p.Thr44Met/p.Lys272Glnfs*14 | DD, SP, hepatic cytolysis, thrombocytopenia, AIHA, autoantibodies, cerebral vascular disease, erythema nodosum of lower limbs, severe polyarthritis, severe aphthous stomatitis, severe hypogammaglobulinaemia, recurrent genital ulcers and abscesses, SS, SD, acute measles infection at age 5 years |
| de Bruin et al. (4) | P26 | F | NA | Yes (P27) | NA | p.Ser258Trpfs*39 (hom) | Severe SS, SD |
|  | P27 | M | NA | Yes (P26) | NA | p.Ser258Trpfs*39 (hom) | Severe SS, SD |
| Briggs et al. (5) | P28 | M | 2 | No | c.821T>C (hom) | p.Val274Ala (hom) | DD, hypothyroidism, infections (severe chickenpox, skin and dental abscesses), SD, SS |
|  | P29 | F | 5 | Yes (P30) | c.643G>A (hom) | p.Gly215Arg (hom) | AITP, cerebral haemorrhage, BC, SS, SD |
|  | P30 | M | 0.33 | Yes (P29) | c.643G>A (hom) | p.Gly215Arg (hom) | Nothing except SD |
|  | P31 | F | 6 | No | c.155A>C (hom) | p.Lys52Thr (hom) | AITP, SD, SS |
|  | P32 | F | 3 | No | c.725A>G (hom) | p.His242Arg (hom) | SS, leg bowing, SD |
|  | P33 | F | 2.5 | Yes (P34) | c.155A>C (hom)/c.790A>G (het) | p.Lys52Thr (hom)/ p.Met264Val (het) | AIHA, SD, SS |
|  | P34 | M | 0.25 | Yes (P33) | c.155A>C (hom)/c.790A>G (het) | p.Lys52Thr (hom)/ p.Met264Val (het) | AIHA, SS, SD |
|  | P35 | F | 2 | No | c.325G>A (hom) | p.Gly109Arg (hom) | SS, SD |
|  | P36 | F | 9 | Yes (P37) | c.389+1G>A (hom) | p.? (hom) | Hepatitis, SD, SS |
|  | P37 | F | 0 | Yes (P36) | c.389+1G>A (hom) | p.? (hom) | AITP leading to death at age 1 year |
|  | P38 | M | 0.08 | No | c.359A>G (hom) | p.Gln120Arg (hom) | AITP, SS, SD, BC |
|  | P39 | M | 15 | No | c.325G>A (hom) | p.Gly109Arg (hom) | SS, SP, SD, BC |
|  | P40 | M | 5 | Yes (P41-43) | c.325G>A/c.712T>C | p.Gly109Arg/p.Cys238Arg | SS, SD |
|  | P41 | F | 0.58 | Yes (P40, 42, 43) | c.325G>A/c.712T>C | p.Gly109Arg/p.Cys238Arg | SS, SD, DD |
|  | P42 | F | 0.5 | Yes (P40, 41, 43) | c.131C>T/c.712T>C | p.Thr44Met/p.Cys238Arg | SS, SD |
|  | P43 | F | 0.5 | Yes (P40-42) | c.131C>T/c.712T>C | p.Thr44Met/p.Cys238Arg | SS, SD |
| Utsumi et al. (6) | P44 | M | 0.42 | No | c.736-2A>C/c.526C>T | ---/p.Arg176* | SS, SD, painful knee joints, BC |
| Sacri et al. (7) | P45 | F | 5 | Yes (brother not in the table) | c.643G>A (hom) | p.Gly215Arg (hom) | AITP, neutropenia, anaemia, SS, SD, SP, normal psychomotor development, BC, ANAs |
|  | P46 | F | 6 | No | c.155A>C (hom) | p.Lys52Thr (hom) | AITP, SS, SD, no neurologic impairment, ANAs, anti-dsDNA |
|  | P47 | M | 3 | No | c.667C>T (hom) | p.Gln223* (hom) | AIHA, SS, SD, SP, normal psychomotor development, BC, ANAs, anti-dsDNA ab |
| Zhong et al. (8) | P48 | F | 2 | No | c.798dupC/c.716G>A | p. Ser267Leufs*20/p.Gly239Asp | DD, SP, recurrent fever, type Ⅱ AIH, SLE, ANAs+, anti-dsDNA ab+, leukopenia, anaemia, mycoplasma pneumonia, suspected fungal infections, SS, SD |
| Lee et al. (9) | P49 | M | 3 | Yes (P50) | c.136C>T/c.449T>A | p.Arg46Trp/p.Val150Glu | Moyamoya, fever, skin lesions suggestive of Raynaud’s phenomenon, leukopenia, ANAs, anti-dsDNA, anticardiolipin antibody IgM, SLE with diffuse proliferative glomerulonephritis, SD |
|  | P50 | F | 8 | Yes (P49) | c.136C>T/c.449T>A | p.Arg46Trp/p.Val150Glu | Moyamoya, SLE, SD |
| Kara et al. (10) | P51 | F | 2 | Yes (P52) | c.155A>C (hom) | p.Lys52Thr (hom) | SS, SD, MAS secondary to sJIA, bilateral parenchymal pulmonary dense infiltrates and mild pleural effusion, AIHA, oral aphthous ulcers, SLE, ANAs, anti-dsDNA, leukopenia, thrombocytopenia, SP, DD |
|  | P52 | M | 19 | Yes (P51) | c.155A>C (hom) | p.Lys52Thr (hom) | SS, SD, SP, mild DD, BC, SLE with class II lupus nephritis, oral aphthous ulcers, ANAs, anti-dsDNA, anti-Sm and Sm/RNP antibodies, gastrointestinal involvement due to SLE |
| Wang et al. (11) | P53 | F | 2 | No | c.798dupC/c.716G>A | p.Ser267Leufs*20/p.Gly239Asp | Fever, nervous system problems, SS, SD, SLE |
| Suri et al. (12) | P54 | F | 13 | No | c.550C>T/c.740T>G | p.Gln184*/p.Leu247Arg | Fever, seizure, stroke, BC, optic atrophy, hypertensive, SS, SLE, IgA nephropathy, ANAs, anti-dsDNA |
|  | P55 | F | 1 | No | c.136delc (hom) | p.Arg46Glyfs*24 (hom) | Fever, anaemia, AITP, facial dysmorphism, DD, BC, SS, SD |
| Aylan Gelen et al. (13) | P56 | M | 5 | No | c.155A>C (hom) | p.Lys52Thr (hom) | AIHA, SS, SD, school academic performance described as 'low', SP, BC, recurrent pneumonia attacks, osteonecrosis of humeri probably due to long-term corticosteroid use |
| Sait et al. (14) | P57 | F | NA | No | c.550C>T/c.740T>G | p.Gln184*/p.Leu247Arg | Seizures due to acute haemorrhagic stroke secondary to malignant hypertension, BC, IgA nephropathy, SS, SD, recurrent bouts of fever, HSM, ANAs, anti-dsDNA, SLE |
| Bağlan et al. (15) | P58 | F | 0.17 | No | c.772_790del (hom) | p.Ser258Trpfs*39 (hom) | DD, BC, recurrent lower respiratory infections, HSM, generalized lymphadenopathy, SS, SD, AIHA, thrombocytopenia, SLE, ANAs, anti-dsDNA, pericardial and pleural effusion |
|  | P59 | F | 01.08 | Yes (P60) | c.155A>C (hom) | p.Lys52Thr (hom) | Severe SS, SD, mild hypogammaglobulinemia, nephrotic syndrome due to membranous nephropathy, autoimmune thyroiditis, BC |
|  | P60 | F | 12.5 | Yes (P59) | c.155A>C (hom) | p.Lys52Thr (hom) | SS, SD, non-autoimmune compensated hypothyroidism |
| Hong et al. (16) | P61 | F | 2 | No | c.1152G>T/c.420G>A | p.Arg46Gln/p.Gly290Val | SLE, AITP, ANAs, anti-dsDNA, SS, no SD and no obvious neurological abnormalities |
| Li et al. (17) | P62 | F | 4 | No | NA | p.Gly215Arg/p.Leu247Pro | SD, recurrent encephalalgia, extrapyramidal symptoms, BC, IgA nephropathy |
|  | P63 | F | 12.67 | No | NA | p.Ser267Leufs*20/p.Gly239Asp | SS, SD, DD, extrapyramidal signs, BC, AIH, liver calcifications, mild bilateral ILD, systemic inflammation, febrile attacks, SS, hypothyroidism |
| Mathiyazhagan et al. (18) | P64 | M | 3 | No | NA | p.Asp203Ala (hom) | AIHA, SP, generalized tonic-clonic seizures, inappropriate laughter and loss of coherent speech, SS, SD, microcephaly, HSM |
| Chougule et al. (19) | P65 | M | 0.17 | No | c.628_634delinsCCTACC (hom) | p.Ser210Profs*48 (hom) | DD, seizures, SP, AIHA and AITP (Evans syndrome), SS, SD |
|  | P66 | F | 0.42 | No | c.325G>A (hom) | p.Gly109Arg (hom) | AIHA and AITP (Evans syndrome), SS, no SD, BC |
| Elhossini et al. (20) | P67 | F | 1.17 | Yes (P68) | c.629C>T (hom) | p.Ser210Phe (hom) | DD, SP, BC, SS, SD, anaemia, thrombocytopenia |
|  | P68 | F | 1.67 | Yes (P67) | c.629C>T (hom) | p.Ser210Phe (hom) | DD, marked SP, SS, SD, anaemia, recurrent skin rash |
|  | P69 | M | 2 | No | c.526C>T (hom) | p.Arg176* (hom) | Seizures, BC, SS, SD, GH deficiency |
|  | P70 | M | 0.33 | No | c.742dupC (hom) | p.Gln248Profs*3 (hom) | DD, SP, BC, frequent attacks of fever, chest infections, SD |
|  | P71 | F | 0.67 | No | c.775G>A (hom) | p.Gly259Arg (hom) | DD, SP, BC, SS, SD, anaemia, neonatal fever, eczema |
| Gernez et al. (21) | P72 | M | 3 | No | c.325G>A/c.526C>T | p.Gly109Arg/p.Arg176* | SS, SD, hypothyroidism (Hashimoto’s disease), AIHA and AITP (Evans syndrome), HSM, ANA, smouldering multiple myeloma, neurologically asymptomatic, BC |
|  | P73 | F | 5 | No | c.733C>T/c.611G>A | p.Gln245*/p.Gly204Asp | SS, SD, DD, BC, HSM, thyroid dysfunction, thyroglobulin antibody, AIHA and AITP (Evans syndrome), neutropenia, SLE, ANAs, anti-dsDNA, myeloid peroxidase (MPO) antibodies |
| Sener et al. (22) | P74 | M | NA | No | c.325G>A (hom) | p.Gly109Arg (hom) | SLE with lupus nephritis, arthritis, leukopenia, SS, SD, fever, ANAs, anti-dsDNA |
| Al-Kateb et al. (23) | P75 | M | 1 | No | c.549del (hom) | p.Gln184Serfs*28 (hom) | AIHA and AITP (Evans syndrome), fever, lower leg pain without arthritis, SP, DD, BC, SS, no SD, GH deficiency, celiac disease, deceased at age 6 years from unexplained sudden respiratory distress |
| Dri et al. (24) | P76 | F | 3 | No | c.791T>A (hom) | p.Met264Lys (hom) | Genu valgum, no SS, SD, no BC |
|  | P77 | F | 6 | No | c.791T>A (hom) | p.Met264Lys (hom) | No SS, SD, DD, SP, BC, recurrent febrile syndrome, septic shock, recurrent pneumonia, ischemic stroke in the context of APL, autoimmune hypothyroidism, thrombocytopenic purpura, AIH |
|  | P78 | F | 2 | No | c.791T>A (hom) | p.Met264Lys (hom) | No SS, SD, polyarthralgia, splenomegaly, cytopenia, fever, lymphoproliferation, SLE with ILD |
|  | P79 | F | 6 | No | c.791T>A/c.632T>C | p.Met264Lys/p.Ile211Thr | SS, SD, DD, BC, humoral immunodeficiency, renal tubular acidosis, AIH, bronchiectasis, septic arthritis of right hip with bacteraemia due to Streptococcus pneumoniae |
| Romano et al. (25) | P80 | F | 0.58 | Yes (sister not in the table) | deletion exons 4–7 (hom) | p.Ex2_5 del (hom) | SS, SD, severe DD, no BC, recurrent lower respiratory infections, HSM, generalized lymphadenopathy, AIHA, thrombocytopenia, SLE, ANAs, anti-dsDNA, pericardial and pleural effusion |
| Firat Senturk et al. (26) | P81 | F | 24 | No | c.155A>C (hom) | p.Lys52Thr (hom) | Immune neutropenia, AITP, autoimmune myelofibrosis, toxoplasma infections of brain tissue and Actinomyces infection of left maxillary sinus, Hashimoto’s thyroiditis, SLE, ANA, anti-dsDNA, significant SS, SD |
| Pekpak Şahinoğlu et al. (27) ^ | P82 | M | NA | No | c.772_790del (hom) | p.Ser258Trpfs*39 (hom) | SS, SD, AIHA, pancytopenia, DD, SP, seizures, BC, hypothyroidism, recurrent respiratory tract infections, deceased at age 9.5 years due to E.coli sepsis |
|  | P83 | F | NA | No | c.772_790del (hom) | p.Ser258Trpfs*39 (hom) | SS, SD, AIHA, DD, hypothyroidism, recurrent respiratory tract infections, ANAs |
|  | P84 | F | NA | Yes (P86) | c.772_790del (hom) | p.Ser258Trpfs*39 (hom) | SS, SD, pancytopenia, HSM, frequent episodes of infection, DD, deceased at age 7 months due to sepsis |
|  | P85 | M | NA | Yes (P89) | c.772_790del (hom) | p.Ser258Trpfs*39 (hom) | SS, SD, AIHA, DD, SP, BC, recurrent respiratory infections |
|  | P86 | F | NA | Yes (P84) | c.772_790del (hom) | p.Ser258Trpfs*39 (hom) | SS, SD, AIHA, DD, hypothyroidism |
|  | P87 | M | NA | No | c.772_790del (hom) | p.Ser258Trpfs*39 (hom) | SS, SD, AITP, DD, SP, BC, hypothyroidism, recurrent respiratory tract infections, ANAs, p-ANCA |
|  | P88 | M | NA | No | c.772_790del (hom) | p.Ser258Trpfs*39 (hom) | SS, SD, AIHA, DD, SP, BC, hypothyroidism |
|  | P89 | M | NA | Yes (P85) | c.772_790del (hom) | p.Ser258Trpfs*39 (hom) | SS, SD, AITP, DD, SP, BC, recurrent respiratory tract infections |
|  | P90 | M | NA | No | c.772_790del (hom) | p.Ser258Trpfs*39 (hom) | SS, SD, AITP, DD, ANAs |

* Cases from the following papers were excluded: Bilginer et al. (28) and Kaya Akca et al. (29) because the genotype is not reported; Shimizu et al. (30) because the genotype is not precisely defined; Wang et al. (31) because the article is in Chinese; Hong et al. (32) because we believe that these patients are the same as reported in Lee et al. (9).

^^^ The patients in Pekpak Şahinoğlu et al. (27) all derive from the same small village and were born to consanguineous parents

AIH: autoimmune hepatitis; AIHA: autoimmune haemolytic anaemia; AITP: autoimmune idiopathic thrombocytopenia; ANAs: antinuclear autoantibody; Anti-dsDNA: anti-double-stranded DNA antibody; APL: antiphospholipid syndrome; BC: brain calcifications; DD: developmental delay; F: female; GH: growth hormone; GI: gastrointestinal; HCL: hydroxychloroquine; Het: heterozygous; Hom: homozygous; HSM: hepatosplenomegaly; IFN: interferon; ILD: interstitial lung disease; IVIG: intravenous immunoglobulin; M: male; MAS: macrophage activation syndrome; MMF: mycophenolate mofetil; NA: not annotated; p-ANCA: perinuclear anti-neutrophil cytoplasmic antibody; SD: skeletal dysplasia; sJIA: systemic juvenile idiopathic arthritis; SLE: systemic lupus erythematosus; SP; spastic paraparesis; SS: short stature
