# Supplementary material for: Spondyloenchondrodysplasia: An enigmatic immuno-osseus type I interferonopathy
Source: J Hum Immun. 2025 Jun 4;1(2):e20250035. doi: 10.70962/jhi.20250035 (PMC7618195; doi:10.70962/jhi.20250035)
Supplement: Table S2 — shows the information on individual mutations recorded in the 27 references noted in Table 1. [file jhi_20250035_tables2.docx]

**Supplementary Table 2. Information on individual mutations recorded in the 27 references noted in Table 1**

| **Mutation** | **Number of pedigrees** | **Mutation type** | **Patient country of origin** |
| --- | --- | --- | --- |
| p.Ex2_5 del (hom) | 2 | deletion | France/Syrian |
| p.Ex3_5 del (hom) | 1 | deletion | India |
| p.Thr44Met | 2 | missense | China/NA |
| p.Arg46Glyfs*24 (hom) | 1 | frameshift | India |
| p.Arg46Trp | 1 | missense | NA |
| p.Arg46Gln | 1 | missense | NA |
| **p.Lys52Thr (hom)** | **8** | **missense** | **Turkey/NA** |
| p.Met64Lys (hom) | 1 | missense | Portugal |
| p.Tyr74* (hom) | 1 | stop | NA |
| p.Thr89Ile (hom) | 1 | missense | Turkey |
| **p.Gly109Arg** | **9** | **missense** | **Iraq/Israel/China/NA** |
| p.Gln120Arg (hom) | 1 | missense | Italy |
| p.Tyr123* | 1 | stop | Austria |
| p.Val150Glu | 1 | missense | NA |
| **p.Arg176*** | **3** | **stop** | **Egypt/Japan** |
| p.Gln184* | 2 | stop | India |
| p.Gln184Serfs*28 (hom) | 1 | frameshift | NA |
| p.Leu201Pro | 1 | missense | NA |
| p.Asp203Ala (hom) | 1 | missense | NA |
| p.Gly204Asp | 1 | missense | NA |
| p.Tyr206* (hom) | 1 | stop | NA |
| p.Ser210Phe (hom) | 1 | missense | Egypt |
| p.Ser210Profs*48 (hom) | 1 | frameshift | NA |
| p.Ile211Thr | 1 | missense | NA |
| **p.Gly215Arg** | **6** | **missense** | **Senegal/Mali/NA** |
| p.Gln223* (hom) | 2 | stop | Pakistan |
| p.Cys238Arg | 2 | missense | China |
| **p.Gly239Asp** | **3** | **missense** | **NA** |
| p.Asp241Asn | 1 | missense | Austria |
| p.His242Arg (hom) | 1 | missense | Mexico |
| p.Gln245* | 1 | stop | NA |
| p.Leu247Arg | 2 | missense | India |
| p.Leu247Pro | 1 | missense | NA |
| p.Gln248Profs*3 (hom) | 1 | frameshift | Egypt |
| **p.Ser258Trpfs*39 (hom)** | **10#** | **frameshift** | **Turkey/Egypt** |
| p.Gly259Arg (hom) | 1 | missense | Egypt |
| p.Asn262His (hom) | 1 | missense | NA |
| **p.Met264Lys** | **5** | **missense** | **NA** |
| p.Met264Val | 1 | missense | Turkey |
| p.Ser267* (hom) | 1 | stop | NA |
| **p.Ser267Leufs*20** | **3** | **frameshift** | **NA** |
| p.Lys272Glnfs*14 | 1 | frameshift | NA |
| p.Val274Ala (hom) | 1 | missense | France |
| p.Tyr278del | 1 | missense | NA |
| p.Gly290Val | 1 | missense | NA |

NA: not annotated

Bold type denotes mutations seen in three or more pedigrees

# Seven pedigrees originating from the same small village (27)

(hom) denotes mutations seen in the homozygous state
