# Supplementary material for: Spondyloenchondrodysplasia: An enigmatic immuno-osseus type I interferonopathy
Source: J Hum Immun. 2025 Jun 4;1(2):e20250035. doi: 10.70962/jhi.20250035 (PMC7618195; doi:10.70962/jhi.20250035)
Supplement: Table S3 — shows the data provided on patients recorded in Table 1 treated using JAK inhibition. [file jhi_20250035_tables3.docx]

**Supplementary Table 3. Data provided on patients recorded in Table 1 treated using JAK inhibition**

| **Reference PMID** | **Patient number in Supplementary Table 1** | **Phenotype** | **JAK inhibitor data** | **Treatment response** |
| --- | --- | --- | --- | --- |
| Wang et al. (11) | P53 | Fever, nervous system problems, SS, SLE | Specific JAK inhibitor used not stated | Not mentioned |
| Chougule et al. (19) | P65 | DD, seizures, SP, AIHA and AITP (Evans syndrome), SS, SD | Already on azathioprine, added ruxolitinib at 0.4 mg/kg/day single daily dose at age 4 years, increased to 0.8 mg/kg/day twice daily (total dose = 10 mg/ day), switched over to baricitinib at 0.1 mg/kg/day increasing to 0.2 mg/kg/day | Improvement in AIHA and gained new milestones under JAK 1/2 inhibition |
|  | P66 | AIHA and AITP (Evans syndrome), SS, no SD, BC | Steroids, 2 g/kg intravenous immunoglobulins, rituximab and oral azathioprine. Started on baricitinib at 0.08 mg/kg/day | Improvement in AIHA and gained new milestones under JAK 1/2 inhibition |
| Gernez et al. (21) | P72 | SLE with lupus nephritis, arthritis, leukopenia, SS, fever, ANAs+, anti-dsDNA ab+ | IVIG, rituximab, sirolimus, bortezomib and MMF and prolonged courses of oral steroids failed to improve his cytopenia. Ruxolitinib was initiated at 0.4 mg/kg/day | Significantly improved energy levels within a week. Very good response of blood indices (anaemia and thrombocytopenia brought into remission within 3 months) to ruxolitinib, without any other additional therapies for the following three-and-a-half years |
|  | P73 | AIHA and AITP (Evans syndrome), fever, lower leg pain without arthritis, SP, DD, SS, growth hormone deficiency, celiac disease, deceased at 6 years old from unexplained sudden respiratory distress | Cytopenias moderately responsive to glucocorticoid therapy, but refractory to rituximab and IVIG, then treated by glucocorticoids and MMF. Ruxolitinib was initiated at 0.4 mg/kg/day | Significantly improved energy levels within one week. Very good response of blood indices (improved platelet counts within one month) to baricitinib, steroid-free for the next 2.5 years, but acute respiratory failure due to parainfluenza and rhinoviral pneumonia with bacterial superinfection and sepsis --> ruxolitinib discontinued and acute flare of ITP treated with glucocorticoids. After resolution of sepsis and following the reinitiation of ruxolitinib therapy, the patient’s platelet count improved within 4 weeks |
| Dri et al. (24) | P77 | No SS, SD, intellectual disability, SP, BC, recurrent febrile syndrome, septic shock, recurrent pneumonia, ischemic stroke in the context of APL, autoimmune hypothyroidism, thrombocytopenic purpura, AIH | Currently being treated with IVIG, ruxolitinib due to a refractory response to first-line immunosuppressants, and HCL with acetylsalicylic acid for APL | Partial response due to an intra-treatment relapse of cytopenia |
|  | P78 | No SS, SD, polyarthralgia, splenomegaly, cytopenia, fever, lymphoproliferation, SLE with ILD | Currently receiving ruxolitinib together with MMF and HCL | Some response noted (“adequate response”) |
| Li et al. (17) | P62 | SD, recurrent encephalalgia, extrapyramidal symptoms, BC, IgA nephropathy | Tofacitinib (0.24 mg/kg/day) | Cessation of headaches after treatment with tofacitinib |
|  | P63 | SS, SD, DD, extrapyramidal features, BC, AIH, liver calcifications, mild bilateral ILD, systemic inflammation, febrile attacks, SS, hypothyroidism | Tofacitinib (0.22 mg/ kg/day) | Febrile attacks reduced after treatment with tofacitinib |
| Firat Senturk et al. (26) | P81 | Immune neutropenia, AITP, autoimmune myelofibrosis, toxoplasma infection in her brain tissue and Actinomyces infection in her left maxillary sinus, Hashimoto’s thyroiditis, SLE, ANAs, anti-dsDNA ab+, significant SS, SD | After trimethoprim/ sulfamethoxazole and HCL, started using baricitinib | Reduction in fatigue levels, positive impact on blood cell counts |
| Romano et al. (25) | P80 | SS, SD, severe DD, no BC, recurrent lower respiratory infections, HSM, generalized lymphadenopathy, AIHA, thrombocytopenia, SLE, ANAs+, anti-dsDNA ab+, pericardial and pleural effusion | Baricitinib (0.5 mg/kg/daily) at the age of 18 months | IFN score decreased after 10 months of baricitinib treatment, the benefit of baricitinib on the neurological aspects of her disease is less clear, with no further neurologic gains observed |

AIH: autoimmune hepatitis; AIHA: autoimmune haemolytic anaemia; AITP: autoimmune idiopathic thrombocytopenia; ANAs: antinuclear autoantibody; Anti-dsDNA: anti-double-stranded DNA antibody; APL: antiphospholipid syndrome; BC: brain calcifications; DD: developmental delay; HCL: hydroxychloroquine; HSM: hepatosplenomegaly; IFN: interferon; ILD: interstitial lung disease; IVIG: intravenous immunoglobulin; MAS: macrophage activation syndrome; MMF: mycophenolate mofetil; p-ANCA: perinuclear anti-neutrophil cytoplasmic antibody; SD: skeletal dysplasia; sJIA: systemic juvenile idiopathic arthritis; SLE: systemic lupus erythematosus; SP; spastic paraparesis; SS: short stature
