# Supplementary material for: Spondyloenchondrodysplasia: An enigmatic immuno-osseus type I interferonopathy
Source: J Hum Immun. Author manuscript; Available in PMC 2025 Oct 4. (PMC7618195; doi:10.70962/jhi.20250035)
Supplement: Supplementary file 1 [file EMS207108-supplement-Supplementary.docx]

**Spondyloenchondrodysplasia: an enigmatic immuno-osseus type I interferonopathy**

Callie C. Y. Wong^1^^, Tifenn Wauquier^2^^, Carolina Uggenti^1^, Colin Stok^1^, Alice Lepelley^2^, Marie-Louise Frémond^2,3,4^, Yanick J Crow^1,2^

**Supplementary information**

**Supplementary Table 1. Summarised demographic, genetic and clinical data available for 90 molecularly proven cases of SPENCD described in 27 reports in the literature***

| **PMID** | **Patient number** | **Sex** | **Age at onset (years)** | **Siblings** | **Mutation(s)** |  | **Phenotype** |
| --- | --- | --- | --- | --- | --- | --- | --- |
|  |  |  |  |  | Nucleotide | Amino acid |  |
| Lausch et al. (1) | P1 | M | 9.5 | No | c.325G>A/c.831_833delCTA | p.Gly109Arg/p.Tyr278del | SS, SD, SLE, APL, steroid-dependent neutropenia, thrombocytopenia, no neurologic signs, normal mental development |
|  | P2 | NA | 1 | Yes (P3) | c.800delC (hom) | p.Ser267* (hom) | SS, SD, frequent fevers, granulocytopenia, ANA, SP, mild DD |
|  | P3 | NA | 12 | Yes (P2) | c.800delC (hom) | p.Ser267* (hom) | SS, SD, granulocytopenia, ANA, no neurologic signs, normal development |
|  | P4 | NA | 3 | No | c.325G>A/c.602T>C | p.Gly109Arg/p.Leu201Pro | Borderline SS, SD, thrombocytopenia, leukopenia, HSM, ANA, BC, SP, normal development |
|  | P5 | NA | 12 | No | c.791T>A (hom) | p.Met264Lys (hom) | SS, SD, frequent fevers, HSM, SLE, ANAs+, no neurologic signs, normal development |
|  | P6 | NA | 4 | No | c.222C>A (hom) | p.Tyr74* (hom) | Mild SS, SD, chronic thrombocytopenia, no neurologic signs, normal development |
|  | P7 | NA | 4 | No | c.784A>C (hom) | p.Asn262His (hom) | Marked SS, SD, AIHA, optic neuritis, ataxia, hemiplegia attributed to vascular infarction or multiple sclerosis, BC, DD |
|  | P8 | F | 16 | Yes (P9) | c.325G>A (hom) | p.Gly109Arg (hom) | SS, SD, SLE with class V lupus nephritis, arthralgia, vitiligo, no neurological signs, BC, mild DD |
|  | P9 | M | 5 | Yes (P8) | c.325G>A (hom) | p.Gly109Arg (hom) | SS, SD, arthritis, ANA, SLE with class IV lupus nephritis, no neurological signs, BC |
|  | P10 | NA | 7 | No | c.643G>A (hom) | p.Gly215Arg (hom) | SS, SD, no extra-skeletal signs, no neurological signs, BC |
|  | P11 | NA | 14 | No | c.643G>A (hom) | p.Gly215Arg (hom) | Moderate SS, SD, chronic thrombocytopenia, ataxia, BC |
|  | P12 | NA | 0.83 | No | c.155A>C (hom) | p.Lys52Thr (hom) | Marked SS, SD, recurrent fever, Kawasaki-like disease, thrombocytopenia and haemolytic anaemia, SLE, ANA, vitiligo, hypothyroidism, SP, seizures, microcephaly, DD |
|  | P13 | NA | 12 | Yes (P14) | c.618C>A (hom) | p.Tyr206* (hom) | Marked SS, SD, rheumatic fever with Sydenham’s chorea |
|  | P14 | NA | 13 | Yes (P13) | c.618C>A (hom) | p.Tyr206* (hom) | SS, SD |
| Briggs et al. (2) | P15 | F | 3 | No | 11,544,822–11,556,767 del (hom) | p.Ex2_5 del (hom) | SD, hypothyroidism, Raynaud's/vasculitis, Sjögren's syndrome, inflammatory myositis, seizures, ANA, deceased at age 30 years due to GI bleeding, heart failure and severe hypertension |
|  | P16 | F | 1 | Yes (P17) | c.369C>A/c.721G>A | p.Tyr123*/p.Asp241Asn | SD, thrombocytopenia, SLE with lupus nephritis, hypothyroidism, DD, ANAs, anti-dsDNA |
|  | P17 | M | 3 | Yes (P16) | c.369C>A/c.721G>A | p.Tyr123*/p.Asp241Asn | SD, AITP |
|  | P18 | M | 2 | Yes (P19) | c. 266C>T (hom) | p.Thr89Ile (hom) | SD, Raynaud's/vasculitis, SP, vasculitic skin rash, ANA, anti-dsDNA |
|  | P19 | F | 14 | Yes (P18) | c. 266C>T (hom) | p.Thr89Ile (hom) | SS, SD, Raynaud's/vasculitis |
|  | P20 | M | 0.67 | No | c.667C>T (hom) | p.Gln223* (hom) | SS, SD, AIHA, ANAs, anti-dsDNA |
|  | P21 | M | 2 | No | 11,543,690–11,548,656 del (hom) | p.Ex3_5 del (hom) | SD, recurrent infections (including pneumonia), thrombocytopenia, SLE with lupus nephritis, ANAs, anti-dsDNA |
|  | P22 | F | 3 | No | c.791 T>A (hom) | p.Met64Lys (hom) | SD, recurrent infections (including pneumonia), SLE, hypothyroidism, thrombocytopenia, non-erosive arthropathy, ANA, anti-dsDNA |
|  | P23 | F | 6 | No | c.643 G>A (hom) | p.Gly215Arg (hom) | SD, thrombocytopenia, AIHA, SLE with lupus nephritis, ANAs, anti-dsDNA |
|  | P24 | F | 4 | No | c.772-790del (hom) | p.Ser258Trpfs*39 (hom) | SD, leg pain, ANAs, anti-dsDNA |
| Girschick et al. (3) | P25 | F | 0.5 | No | c.131C>T/c.816dupC | p.Thr44Met/p.Lys272Glnfs*14 | DD, SP, hepatic cytolysis, thrombocytopenia, AIHA, autoantibodies, cerebral vascular disease, erythema nodosum of lower limbs, severe polyarthritis, severe aphthous stomatitis, severe hypogammaglobulinaemia, recurrent genital ulcers and abscesses, SS, SD, acute measles infection at age 5 years |
| de Bruin et al. (4) | P26 | F | NA | Yes (P27) | NA | p.Ser258Trpfs*39 (hom) | Severe SS, SD |
|  | P27 | M | NA | Yes (P26) | NA | p.Ser258Trpfs*39 (hom) | Severe SS, SD |
| Briggs et al. (5) | P28 | M | 2 | No | c.821T>C (hom) | p.Val274Ala (hom) | DD, hypothyroidism, infections (severe chickenpox, skin and dental abscesses), SD, SS |
|  | P29 | F | 5 | Yes (P30) | c.643G>A (hom) | p.Gly215Arg (hom) | AITP, cerebral haemorrhage, BC, SS, SD |
|  | P30 | M | 0.33 | Yes (P29) | c.643G>A (hom) | p.Gly215Arg (hom) | Nothing except SD |
|  | P31 | F | 6 | No | c.155A>C (hom) | p.Lys52Thr (hom) | AITP, SD, SS |
|  | P32 | F | 3 | No | c.725A>G (hom) | p.His242Arg (hom) | SS, leg bowing, SD |
|  | P33 | F | 2.5 | Yes (P34) | c.155A>C (hom)/c.790A>G (het) | p.Lys52Thr (hom)/ p.Met264Val (het) | AIHA, SD, SS |
|  | P34 | M | 0.25 | Yes (P33) | c.155A>C (hom)/c.790A>G (het) | p.Lys52Thr (hom)/ p.Met264Val (het) | AIHA, SS, SD |
|  | P35 | F | 2 | No | c.325G>A (hom) | p.Gly109Arg (hom) | SS, SD |
|  | P36 | F | 9 | Yes (P37) | c.389+1G>A (hom) | p.? (hom) | Hepatitis, SD, SS |
|  | P37 | F | 0 | Yes (P36) | c.389+1G>A (hom) | p.? (hom) | AITP leading to death at age 1 year |
|  | P38 | M | 0.08 | No | c.359A>G (hom) | p.Gln120Arg (hom) | AITP, SS, SD, BC |
|  | P39 | M | 15 | No | c.325G>A (hom) | p.Gly109Arg (hom) | SS, SP, SD, BC |
|  | P40 | M | 5 | Yes (P41-43) | c.325G>A/c.712T>C | p.Gly109Arg/p.Cys238Arg | SS, SD |
|  | P41 | F | 0.58 | Yes (P40, 42, 43) | c.325G>A/c.712T>C | p.Gly109Arg/p.Cys238Arg | SS, SD, DD |
|  | P42 | F | 0.5 | Yes (P40, 41, 43) | c.131C>T/c.712T>C | p.Thr44Met/p.Cys238Arg | SS, SD |
|  | P43 | F | 0.5 | Yes (P40-42) | c.131C>T/c.712T>C | p.Thr44Met/p.Cys238Arg | SS, SD |
| Utsumi et al. (6) | P44 | M | 0.42 | No | c.736-2A>C/c.526C>T | ---/p.Arg176* | SS, SD, painful knee joints, BC |
| Sacri et al. (7) | P45 | F | 5 | Yes (brother not in the table) | c.643G>A (hom) | p.Gly215Arg (hom) | AITP, neutropenia, anaemia, SS, SD, SP, normal psychomotor development, BC, ANAs |
|  | P46 | F | 6 | No | c.155A>C (hom) | p.Lys52Thr (hom) | AITP, SS, SD, no neurologic impairment, ANAs, anti-dsDNA |
|  | P47 | M | 3 | No | c.667C>T (hom) | p.Gln223* (hom) | AIHA, SS, SD, SP, normal psychomotor development, BC, ANAs, anti-dsDNA ab |
| Zhong et al. (8) | P48 | F | 2 | No | c.798dupC/c.716G>A | p. Ser267Leufs*20/p.Gly239Asp | DD, SP, recurrent fever, type Ⅱ AIH, SLE, ANAs+, anti-dsDNA ab+, leukopenia, anaemia, mycoplasma pneumonia, suspected fungal infections, SS, SD |
| Lee et al. (9) | P49 | M | 3 | Yes (P50) | c.136C>T/c.449T>A | p.Arg46Trp/p.Val150Glu | Moyamoya, fever, skin lesions suggestive of Raynaud’s phenomenon, leukopenia, ANAs, anti-dsDNA, anticardiolipin antibody IgM, SLE with diffuse proliferative glomerulonephritis, SD |
|  | P50 | F | 8 | Yes (P49) | c.136C>T/c.449T>A | p.Arg46Trp/p.Val150Glu | Moyamoya, SLE, SD |
| Kara et al. (10) | P51 | F | 2 | Yes (P52) | c.155A>C (hom) | p.Lys52Thr (hom) | SS, SD, MAS secondary to sJIA, bilateral parenchymal pulmonary dense infiltrates and mild pleural effusion, AIHA, oral aphthous ulcers, SLE, ANAs, anti-dsDNA, leukopenia, thrombocytopenia, SP, DD |
|  | P52 | M | 19 | Yes (P51) | c.155A>C (hom) | p.Lys52Thr (hom) | SS, SD, SP, mild DD, BC, SLE with class II lupus nephritis, oral aphthous ulcers, ANAs, anti-dsDNA, anti-Sm and Sm/RNP antibodies, gastrointestinal involvement due to SLE |
| Wang et al. (11) | P53 | F | 2 | No | c.798dupC/c.716G>A | p.Ser267Leufs*20/p.Gly239Asp | Fever, nervous system problems, SS, SD, SLE |
| Suri et al. (12) | P54 | F | 13 | No | c.550C>T/c.740T>G | p.Gln184*/p.Leu247Arg | Fever, seizure, stroke, BC, optic atrophy, hypertensive, SS, SLE, IgA nephropathy, ANAs, anti-dsDNA |
|  | P55 | F | 1 | No | c.136delc (hom) | p.Arg46Glyfs*24 (hom) | Fever, anaemia, AITP, facial dysmorphism, DD, BC, SS, SD |
| Aylan Gelen et al. (13) | P56 | M | 5 | No | c.155A>C (hom) | p.Lys52Thr (hom) | AIHA, SS, SD, school academic performance described as 'low', SP, BC, recurrent pneumonia attacks, osteonecrosis of humeri probably due to long-term corticosteroid use |
| Sait et al. (14) | P57 | F | NA | No | c.550C>T/c.740T>G | p.Gln184*/p.Leu247Arg | Seizures due to acute haemorrhagic stroke secondary to malignant hypertension, BC, IgA nephropathy, SS, SD, recurrent bouts of fever, HSM, ANAs, anti-dsDNA, SLE |
| Bağlan et al. (15) | P58 | F | 0.17 | No | c.772_790del (hom) | p.Ser258Trpfs*39 (hom) | DD, BC, recurrent lower respiratory infections, HSM, generalized lymphadenopathy, SS, SD, AIHA, thrombocytopenia, SLE, ANAs, anti-dsDNA, pericardial and pleural effusion |
|  | P59 | F | 01.08 | Yes (P60) | c.155A>C (hom) | p.Lys52Thr (hom) | Severe SS, SD, mild hypogammaglobulinemia, nephrotic syndrome due to membranous nephropathy, autoimmune thyroiditis, BC |
|  | P60 | F | 12.5 | Yes (P59) | c.155A>C (hom) | p.Lys52Thr (hom) | SS, SD, non-autoimmune compensated hypothyroidism |
| Hong et al. (16) | P61 | F | 2 | No | c.1152G>T/c.420G>A | p.Arg46Gln/p.Gly290Val | SLE, AITP, ANAs, anti-dsDNA, SS, no SD and no obvious neurological abnormalities |
| Li et al. (17) | P62 | F | 4 | No | NA | p.Gly215Arg/p.Leu247Pro | SD, recurrent encephalalgia, extrapyramidal symptoms, BC, IgA nephropathy |
|  | P63 | F | 12.67 | No | NA | p.Ser267Leufs*20/p.Gly239Asp | SS, SD, DD, extrapyramidal signs, BC, AIH, liver calcifications, mild bilateral ILD, systemic inflammation, febrile attacks, SS, hypothyroidism |
| Mathiyazhagan et al. (18) | P64 | M | 3 | No | NA | p.Asp203Ala (hom) | AIHA, SP, generalized tonic-clonic seizures, inappropriate laughter and loss of coherent speech, SS, SD, microcephaly, HSM |
| Chougule et al. (19) | P65 | M | 0.17 | No | c.628_634delinsCCTACC (hom) | p.Ser210Profs*48 (hom) | DD, seizures, SP, AIHA and AITP (Evans syndrome), SS, SD |
|  | P66 | F | 0.42 | No | c.325G>A (hom) | p.Gly109Arg (hom) | AIHA and AITP (Evans syndrome), SS, no SD, BC |
| Elhossini et al. (20) | P67 | F | 1.17 | Yes (P68) | c.629C>T (hom) | p.Ser210Phe (hom) | DD, SP, BC, SS, SD, anaemia, thrombocytopenia |
|  | P68 | F | 1.67 | Yes (P67) | c.629C>T (hom) | p.Ser210Phe (hom) | DD, marked SP, SS, SD, anaemia, recurrent skin rash |
|  | P69 | M | 2 | No | c.526C>T (hom) | p.Arg176* (hom) | Seizures, BC, SS, SD, GH deficiency |
|  | P70 | M | 0.33 | No | c.742dupC (hom) | p.Gln248Profs*3 (hom) | DD, SP, BC, frequent attacks of fever, chest infections, SD |
|  | P71 | F | 0.67 | No | c.775G>A (hom) | p.Gly259Arg (hom) | DD, SP, BC, SS, SD, anaemia, neonatal fever, eczema |
| Gernez et al. (21) | P72 | M | 3 | No | c.325G>A/c.526C>T | p.Gly109Arg/p.Arg176* | SS, SD, hypothyroidism (Hashimoto’s disease), AIHA and AITP (Evans syndrome), HSM, ANA, smouldering multiple myeloma, neurologically asymptomatic, BC |
|  | P73 | F | 5 | No | c.733C>T/c.611G>A | p.Gln245*/p.Gly204Asp | SS, SD, DD, BC, HSM, thyroid dysfunction, thyroglobulin antibody, AIHA and AITP (Evans syndrome), neutropenia, SLE, ANAs, anti-dsDNA, myeloid peroxidase (MPO) antibodies |
| Sener et al. (22) | P74 | M | NA | No | c.325G>A (hom) | p.Gly109Arg (hom) | SLE with lupus nephritis, arthritis, leukopenia, SS, SD, fever, ANAs, anti-dsDNA |
| Al-Kateb et al. (23) | P75 | M | 1 | No | c.549del (hom) | p.Gln184Serfs*28 (hom) | AIHA and AITP (Evans syndrome), fever, lower leg pain without arthritis, SP, DD, BC, SS, no SD, GH deficiency, celiac disease, deceased at age 6 years from unexplained sudden respiratory distress |
| Dri et al. (24) | P76 | F | 3 | No | c.791T>A (hom) | p.Met264Lys (hom) | Genu valgum, no SS, SD, no BC |
|  | P77 | F | 6 | No | c.791T>A (hom) | p.Met264Lys (hom) | No SS, SD, DD, SP, BC, recurrent febrile syndrome, septic shock, recurrent pneumonia, ischemic stroke in the context of APL, autoimmune hypothyroidism, thrombocytopenic purpura, AIH |
|  | P78 | F | 2 | No | c.791T>A (hom) | p.Met264Lys (hom) | No SS, SD, polyarthralgia, splenomegaly, cytopenia, fever, lymphoproliferation, SLE with ILD |
|  | P79 | F | 6 | No | c.791T>A/c.632T>C | p.Met264Lys/p.Ile211Thr | SS, SD, DD, BC, humoral immunodeficiency, renal tubular acidosis, AIH, bronchiectasis, septic arthritis of right hip with bacteraemia due to Streptococcus pneumoniae |
| Romano et al. (25) | P80 | F | 0.58 | Yes (sister not in the table) | deletion exons 4–7 (hom) | p.Ex2_5 del (hom) | SS, SD, severe DD, no BC, recurrent lower respiratory infections, HSM, generalized lymphadenopathy, AIHA, thrombocytopenia, SLE, ANAs, anti-dsDNA, pericardial and pleural effusion |
| Firat Senturk et al. (26) | P81 | F | 24 | No | c.155A>C (hom) | p.Lys52Thr (hom) | Immune neutropenia, AITP, autoimmune myelofibrosis, toxoplasma infections of brain tissue and Actinomyces infection of left maxillary sinus, Hashimoto’s thyroiditis, SLE, ANA, anti-dsDNA, significant SS, SD |
| Pekpak Şahinoğlu et al. (27) ^ | P82 | M | NA | No | c.772_790del (hom) | p.Ser258Trpfs*39 (hom) | SS, SD, AIHA, pancytopenia, DD, SP, seizures, BC, hypothyroidism, recurrent respiratory tract infections, deceased at age 9.5 years due to E.coli sepsis |
|  | P83 | F | NA | No | c.772_790del (hom) | p.Ser258Trpfs*39 (hom) | SS, SD, AIHA, DD, hypothyroidism, recurrent respiratory tract infections, ANAs |
|  | P84 | F | NA | Yes (P86) | c.772_790del (hom) | p.Ser258Trpfs*39 (hom) | SS, SD, pancytopenia, HSM, frequent episodes of infection, DD, deceased at age 7 months due to sepsis |
|  | P85 | M | NA | Yes (P89) | c.772_790del (hom) | p.Ser258Trpfs*39 (hom) | SS, SD, AIHA, DD, SP, BC, recurrent respiratory infections |
|  | P86 | F | NA | Yes (P84) | c.772_790del (hom) | p.Ser258Trpfs*39 (hom) | SS, SD, AIHA, DD, hypothyroidism |
|  | P87 | M | NA | No | c.772_790del (hom) | p.Ser258Trpfs*39 (hom) | SS, SD, AITP, DD, SP, BC, hypothyroidism, recurrent respiratory tract infections, ANAs, p-ANCA |
|  | P88 | M | NA | No | c.772_790del (hom) | p.Ser258Trpfs*39 (hom) | SS, SD, AIHA, DD, SP, BC, hypothyroidism |
|  | P89 | M | NA | Yes (P85) | c.772_790del (hom) | p.Ser258Trpfs*39 (hom) | SS, SD, AITP, DD, SP, BC, recurrent respiratory tract infections |
|  | P90 | M | NA | No | c.772_790del (hom) | p.Ser258Trpfs*39 (hom) | SS, SD, AITP, DD, ANAs |

* Cases from the following papers were excluded: Bilginer et al. (28) and Kaya Akca et al. (29) because the genotype is not reported; Shimizu et al. (30) because the genotype is not precisely defined; Wang et al. (31) because the article is in Chinese; Hong et al. (32) because we believe that these patients are the same as reported in Lee et al. (9).

^^ ^The patients in Pekpak Şahinoğlu et al. (27) all derive from the same small village and were born to consanguineous parents

AIH: autoimmune hepatitis; AIHA: autoimmune haemolytic anaemia; AITP: autoimmune idiopathic thrombocytopenia; ANAs: antinuclear autoantibody; Anti-dsDNA: anti-double-stranded DNA antibody; APL: antiphospholipid syndrome; BC: brain calcifications; DD: developmental delay; F: female; GH: growth hormone; GI: gastrointestinal; HCL: hydroxychloroquine; Het: heterozygous; Hom: homozygous; HSM: hepatosplenomegaly; IFN: interferon; ILD: interstitial lung disease; IVIG: intravenous immunoglobulin; M: male; MAS: macrophage activation syndrome; MMF: mycophenolate mofetil; NA: not annotated; p-ANCA: perinuclear anti-neutrophil cytoplasmic antibody; SD: skeletal dysplasia; sJIA: systemic juvenile idiopathic arthritis; SLE: systemic lupus erythematosus; SP; spastic paraparesis; SS: short stature

**Supplementary Table 2. Information on individual mutations recorded in the 27 references noted in Table 1**

| **Mutation** | **Number of pedigrees** | **Mutation type** | **Patient country of origin** |
| --- | --- | --- | --- |
| p.Ex2_5 del (hom) | 2 | deletion | France/Syrian |
| p.Ex3_5 del (hom) | 1 | deletion | India |
| p.Thr44Met | 2 | missense | China/NA |
| p.Arg46Glyfs*24 (hom) | 1 | frameshift | India |
| p.Arg46Trp | 1 | missense | NA |
| p.Arg46Gln | 1 | missense | NA |
| **p.Lys52Thr (hom)** | **8** | **missense** | **Turkey/NA** |
| p.Met64Lys (hom) | 1 | missense | Portugal |
| p.Tyr74* (hom) | 1 | stop | NA |
| p.Thr89Ile (hom) | 1 | missense | Turkey |
| **p.Gly109Arg** | **9** | **missense** | **Iraq/Israel/China/NA** |
| p.Gln120Arg (hom) | 1 | missense | Italy |
| p.Tyr123* | 1 | stop | Austria |
| p.Val150Glu | 1 | missense | NA |
| **p.Arg176*** | **3** | **stop** | **Egypt/Japan** |
| p.Gln184* | 2 | stop | India |
| p.Gln184Serfs*28 (hom) | 1 | frameshift | NA |
| p.Leu201Pro | 1 | missense | NA |
| p.Asp203Ala (hom) | 1 | missense | NA |
| p.Gly204Asp | 1 | missense | NA |
| p.Tyr206* (hom) | 1 | stop | NA |
| p.Ser210Phe (hom) | 1 | missense | Egypt |
| p.Ser210Profs*48 (hom) | 1 | frameshift | NA |
| p.Ile211Thr | 1 | missense | NA |
| **p.Gly215Arg** | **6** | **missense** | **Senegal/Mali/NA** |
| p.Gln223* (hom) | 2 | stop | Pakistan |
| p.Cys238Arg | 2 | missense | China |
| **p.Gly239Asp** | **3** | **missense** | **NA** |
| p.Asp241Asn | 1 | missense | Austria |
| p.His242Arg (hom) | 1 | missense | Mexico |
| p.Gln245* | 1 | stop | NA |
| p.Leu247Arg | 2 | missense | India |
| p.Leu247Pro | 1 | missense | NA |
| p.Gln248Profs*3 (hom) | 1 | frameshift | Egypt |
| **p.Ser258Trpfs*39 (hom)** | **10#** | **frameshift** | **Turkey/Egypt** |
| p.Gly259Arg (hom) | 1 | missense | Egypt |
| p.Asn262His (hom) | 1 | missense | NA |
| **p.Met264Lys** | **5** | **missense** | **NA** |
| p.Met264Val | 1 | missense | Turkey |
| p.Ser267* (hom) | 1 | stop | NA |
| **p.Ser267Leufs*20** | **3** | **frameshift** | **NA** |
| p.Lys272Glnfs*14 | 1 | frameshift | NA |
| p.Val274Ala (hom) | 1 | missense | France |
| p.Tyr278del | 1 | missense | NA |
| p.Gly290Val | 1 | missense | NA |

NA: not annotated

Bold type denotes mutations seen in three or more pedigrees

# Seven pedigrees originating from the same small village (27) 

(hom) denotes mutations seen in the homozygous state

**Supplementary Table 3. Data provided on patients recorded in Table 1 treated using JAK inhibition**

| **Reference PMID** | **Patient number in Supplementary Table 1** | **Phenotype** | **JAK inhibitor data** | **Treatment response** |
| --- | --- | --- | --- | --- |
| Wang et al. (11) | P53 | Fever, nervous system problems, SS, SLE | Specific JAK inhibitor used not stated | Not mentioned |
| Chougule et al. (19) | P65 | DD, seizures, SP, AIHA and AITP (Evans syndrome), SS, SD | Already on azathioprine, added ruxolitinib at 0.4 mg/kg/day single daily dose at age 4 years, increased to 0.8 mg/kg/day twice daily (total dose = 10 mg/ day), switched over to baricitinib at 0.1 mg/kg/day increasing to 0.2 mg/kg/day | Improvement in AIHA and gained new milestones under JAK 1/2 inhibition |
|  | P66 | AIHA and AITP (Evans syndrome), SS, no SD, BC | Steroids, 2 g/kg intravenous immunoglobulins, rituximab and oral azathioprine. Started on baricitinib at 0.08 mg/kg/day | Improvement in AIHA and gained new milestones under JAK 1/2 inhibition |
| Gernez et al. (21) | P72 | SLE with lupus nephritis, arthritis, leukopenia, SS, fever, ANAs+, anti-dsDNA ab+ | IVIG, rituximab, sirolimus, bortezomib and MMF and prolonged courses of oral steroids failed to improve his cytopenia. Ruxolitinib was initiated at 0.4 mg/kg/day | Significantly improved energy levels within a week. Very good response of blood indices (anaemia and thrombocytopenia brought into remission within 3 months) to ruxolitinib, without any other additional therapies for the following three-and-a-half years |
|  | P73 | AIHA and AITP (Evans syndrome), fever, lower leg pain without arthritis, SP, DD, SS, growth hormone deficiency, celiac disease, deceased at 6 years old from unexplained sudden respiratory distress | Cytopenias moderately responsive to glucocorticoid therapy, but refractory to rituximab and IVIG, then treated by glucocorticoids and MMF. Ruxolitinib was initiated at 0.4 mg/kg/day | Significantly improved energy levels within one week. Very good response of blood indices (improved platelet counts within one month) to baricitinib, steroid-free for the next 2.5 years, but acute respiratory failure due to parainfluenza and rhinoviral pneumonia with bacterial superinfection and sepsis --> ruxolitinib discontinued and acute flare of ITP treated with glucocorticoids. After resolution of sepsis and following the reinitiation of ruxolitinib therapy, the patient’s platelet count improved within 4 weeks |
| Dri et al. (24) | P77 | No SS, SD, intellectual disability, SP, BC, recurrent febrile syndrome, septic shock, recurrent pneumonia, ischemic stroke in the context of APL, autoimmune hypothyroidism, thrombocytopenic purpura, AIH | Currently being treated with IVIG, ruxolitinib due to a refractory response to first-line immunosuppressants, and HCL with acetylsalicylic acid for APL | Partial response due to an intra-treatment relapse of cytopenia |
|  | P78 | No SS, SD, polyarthralgia, splenomegaly, cytopenia, fever, lymphoproliferation, SLE with ILD | Currently receiving ruxolitinib together with MMF and HCL | Some response noted (“adequate response”) |
| Li et al. (17) | P62 | SD, recurrent encephalalgia, extrapyramidal symptoms, BC, IgA nephropathy | Tofacitinib (0.24 mg/kg/day) | Cessation of headaches after treatment with tofacitinib |
|  | P63 | SS, SD, DD, extrapyramidal features, BC, AIH, liver calcifications, mild bilateral ILD, systemic inflammation, febrile attacks, SS, hypothyroidism | Tofacitinib (0.22 mg/ kg/day) | Febrile attacks reduced after treatment with tofacitinib |
| Firat Senturk et al. (26) | P81 | Immune neutropenia, AITP, autoimmune myelofibrosis, toxoplasma infection in her brain tissue and Actinomyces infection in her left maxillary sinus, Hashimoto’s thyroiditis, SLE, ANAs, anti-dsDNA ab+, significant SS, SD | After trimethoprim/ sulfamethoxazole and HCL, started using baricitinib | Reduction in fatigue levels, positive impact on blood cell counts |
| Romano et al. (25) | P80 | SS, SD, severe DD, no BC, recurrent lower respiratory infections, HSM, generalized lymphadenopathy, AIHA, thrombocytopenia, SLE, ANAs+, anti-dsDNA ab+, pericardial and pleural effusion | Baricitinib (0.5 mg/kg/daily) at the age of 18 months | IFN score decreased after 10 months of baricitinib treatment, the benefit of baricitinib on the neurological aspects of her disease is less clear, with no further neurologic gains observed |

AIH: autoimmune hepatitis; AIHA: autoimmune haemolytic anaemia; AITP: autoimmune idiopathic thrombocytopenia; ANAs: antinuclear autoantibody; Anti-dsDNA: anti-double-stranded DNA antibody; APL: antiphospholipid syndrome; BC: brain calcifications; DD: developmental delay; HCL: hydroxychloroquine; HSM: hepatosplenomegaly; IFN: interferon; ILD: interstitial lung disease; IVIG: intravenous immunoglobulin; MAS: macrophage activation syndrome; MMF: mycophenolate mofetil; p-ANCA: perinuclear anti-neutrophil cytoplasmic antibody; SD: skeletal dysplasia; sJIA: systemic juvenile idiopathic arthritis; SLE: systemic lupus erythematosus; SP; spastic paraparesis; SS: short stature

**Supplementary file references**

1.	Lausch, E., A. Janecke, M. Bros, S. Trojandt, Y. Alanay, C. De Laet, C.A. Hubner, P. Meinecke, G. Nishimura, M. Matsuo, et al. 2011. Genetic deficiency of tartrate-resistant acid phosphatase associated with skeletal dysplasia, cerebral calcifications and autoimmunity. *Nat Genet*. 43(2):132-7. [https://doi.org/10.1038/ng.749](https://doi.org/10.1038/ng.749)

2.	Briggs, T.A., G.I. Rice, S. Daly, J. Urquhart, H. Gornall, B. Bader-Meunier, K. Baskar, S. Baskar, V. Baudouin, M.W. Beresford, et al. 2011. Tartrate-resistant acid phosphatase deficiency causes a bone dysplasia with autoimmunity and a type I interferon expression signature. *Nat Genet*. 43(2):127-31. [https://doi.org/10.1038/ng.748](https://doi.org/10.1038/ng.748)

3.	Girschick, H., C. Wolf, H. Morbach, C. Hertzberg, M.A. Lee-Kirsch. 2015. Severe immune dysregulation with neurological impairment and minor bone changes in a child with spondyloenchondrodysplasia due to two novel mutations in the ACP5 gene. *Pediatr Rheumatol Online J*. 13(1):37. [https://doi.org/10.1186/s12969-015-0035-7](https://doi.org/10.1186/s12969-015-0035-7)

4.	de Bruin, C., Z. Orbak, M. Andrew, V. Hwa, A. Dauber. 2016. Severe Short Stature in Two Siblings as the Presenting Sign of ACP5 Deficiency. *Horm Res Paediatr*. 85(5):358-62. [https://doi.org/10.1159/000443684](https://doi.org/10.1159/000443684)

5.	Briggs, T.A., G.I. Rice, N. Adib, L. Ades, S. Barete, K. Baskar, V. Baudouin, A.N. Cebeci, P. Clapuyt, D. Coman, et al. 2016. Spondyloenchondrodysplasia Due to Mutations in *ACP5*: A Comprehensive Survey. *J Clin Immunol*. 36(3):220-34. [https://doi.org/10.1007/s10875-016-0252-y](https://doi.org/10.1007/s10875-016-0252-y)

6.	Utsumi, T., S. Okada, K. Izawa, Y. Honda, G. Nishimura, R. Nishikomori, R. Okano, M. Kobayashi. 2017. A Case with Spondyloenchondrodysplasia Treated with Growth Hormone. *Front Endocrinol (Lausanne)*. 8:157. [https://doi.org/10.3389/fendo.2017.00157](https://doi.org/10.3389/fendo.2017.00157)

7.	Sacri, A.S., A. Bruwier, G. Baujat, S. Breton, S. Blanche, T.A. Briggs, B. Bader-Meunier. 2017. Childhood-onset autoimmune cytopenia as the presenting feature of biallelic ACP5 mutations. *Pediatr Blood Cancer*. 64(2):306-10. [https://doi.org/10.1002/pbc.26195](https://doi.org/10.1002/pbc.26195)

8.	Zhong, L.Q., L. Wang, H.M. Song, W. Wang, M. Wei, Y.Y. He. 2018. [Spondyloenchondrodysplasia with immune dysregulation: a case report and literature review]. *Zhonghua Er Ke Za Zhi*. 56(8):611-6. [https://doi.org/10.3760/cma.j.issn.0578-1310.2018.08.011](https://doi.org/10.3760/cma.j.issn.0578-1310.2018.08.011)

9.	Lee, J.S., B.C. Lim, K.J. Kim, S.K. Kim, O.H. Kim, J.G. Kim, S.G. Park, T.J. Cho, J.H. Chae. 2019. A Case of Familial Spondyloenchondrodysplasia with Immune Dysregulation Masquerading as Moyamoya Syndrome. *J Clin Neurol*. 15(3):407-9. [https://doi.org/10.3988/jcn.2019.15.3.407](https://doi.org/10.3988/jcn.2019.15.3.407)

10.	Kara, B., Z. Ekinci, S. Sahin, M. Gungor, A.S. Gunes, K. Ozturk, A. Adrovic, A. Cefle, M. Inanç, A. Gul, et al. 2020. Monogenic lupus due to spondyloenchondrodysplasia with spastic paraparesis and intracranial calcification: case-based review. *Rheumatol Int*. 40(11):1903-10. [https://doi.org/10.1007/s00296-020-04653-x](https://doi.org/10.1007/s00296-020-04653-x)

11.	Wang, W., Z. Yu, L. Gou, L. Zhong, J. Li, M. Ma, C. Wang, Y. Zhou, Y. Ru, Z. Sun, et al. 2020. Single-Center Overview of Pediatric Monogenic Autoinflammatory Diseases in the Past Decade: A Summary and Beyond. *Front Immunol*. 11:565099. [https://doi.org/10.3389/fimmu.2020.565099](https://doi.org/10.3389/fimmu.2020.565099)

12.	Suri, D., A. Rawat, A.K. Jindal, P. Vignesh, A. Gupta, R.K. Pilania, V. Joshi, K. Arora, R. Kumrah, G. Anjani, et al. 2021. Spectrum of Systemic Auto-Inflammatory Diseases in India: A Multi-Centric Experience. *Front Immunol*. 12:630691. [https://doi.org/10.3389/fimmu.2021.630691](https://doi.org/10.3389/fimmu.2021.630691)

13.	Aylan Gelen, S., B. Kara, I. Eser Simsek, M. Gungor, E. Zengin, N. Sarper. 2024. Autoimmune Hemolytic Anemia Due to Spondyloenchondrodysplasia with Spastic Paraparesis and Intracranial Calcification due to Mutation in ACP5. *J Pediatr Genet*. 13(1):50-6. [https://doi.org/10.1055/s-0041-1736560](https://doi.org/10.1055/s-0041-1736560)

14.	Sait, H., H. Gangadharan, A. Gupta, A. Aggarwal, M. Jain, S.R. Phadke. 2021. Monogenic Lupus with IgA Nephropathy Caused by Spondyloenchondrodysplasia with Immune Dysregulation. *Indian J Pediatr*. 88(8):819-23. [https://doi.org/10.1007/s12098-020-03636-x](https://doi.org/10.1007/s12098-020-03636-x)

15.	Bağlan, E., S. Özdel, T. Güngör, R. Karakuş, E.Ü. Bağrıaçık, A.A. Yücel, M. Bülbül. 2021. Spondylenchondrodysplasia mimicking a systemic lupus erythematosus: A diagnostic challenge in a pediatric patient. *Eur J Med Genet*. 64(9):104286. [https://doi.org/10.1016/j.ejmg.2021.104286](https://doi.org/10.1016/j.ejmg.2021.104286)

16.	Hong, S.M., W. Chen, J. Feng, D. Dai, N. Shen. 2022. Novel Mutations in ACP5 and SAMHD1 in a Patient With Pediatric Systemic Lupus Erythematosus. *Front Pediatr*. 10:885006. [https://doi.org/10.3389/fped.2022.885006](https://doi.org/10.3389/fped.2022.885006)

17.	Li, W., W. Wang, W. Wang, L. Zhong, L. Gou, C. Wang, J. Ma, M. Quan, S. Jian, X. Tang, et al. 2022. Janus Kinase Inhibitors in the Treatment of Type I Interferonopathies: A Case Series From a Single Center in China. *Front Immunol*. 13:825367. [https://doi.org/10.3389/fimmu.2022.825367](https://doi.org/10.3389/fimmu.2022.825367)

18.	Mathiyazhagan, G., F. Hasan, P. Chauhan, A. Gupta, S. Yadav, R. Kashyap. 2022. Spondyloenchondrodysplasia with immune dysregulation: Role of sirolimus. *Pediatr Blood Cancer*. 69(10):e29672. [https://doi.org/10.1002/pbc.29672](https://doi.org/10.1002/pbc.29672)

19.	Chougule, A., P. Taur, V. Gowri, C.O.E. Consortium, M.M. Desai. 2023. SPENCD Presenting with Evans Phenotype and Clinical Response to JAK1/2 Inhibitors-a Report of 2 Cases. *J Clin Immunol*. 43(2):331-4. [https://doi.org/10.1007/s10875-022-01400-8](https://doi.org/10.1007/s10875-022-01400-8)

20.	Elhossini, R.M., H.M. Elbendary, K. Rafat, R.M. Ghorab, M.S. Abdel-Hamid. 2023. Spondyloenchondrodysplasia in five new patients: identification of three novel ACP5 variants with variable neurological presentations. *Mol Genet Genomics*. 298(3):709-20. [https://doi.org/10.1007/s00438-023-02009-1](https://doi.org/10.1007/s00438-023-02009-1)

21.	Gernez, Y., M. Narula, A.M. Cepika, J. Valdes Camacho, E.G. Hoyte, K. Mouradian, B. Glader, D. Singh, B. Sathi, L. Rao, et al. 2023. Case report: Refractory Evans syndrome in two patients with spondyloenchondrodysplasia with immune dysregulation treated successfully with JAK1/JAK2 inhibition. *Front Immunol*. 14:1328005. [https://doi.org/10.3389/fimmu.2023.1328005](https://doi.org/10.3389/fimmu.2023.1328005)

22.	Sener, S., E. Sag, X. Han, Y. Bilginer, Q. Zhou, S. Ozen. 2024. Detection of genetic mutations underlying early-onset systemic lupus erythematosus. *Lupus*. 33(9):998-1003. [https://doi.org/10.1177/09612033241255011](https://doi.org/10.1177/09612033241255011)

23.	Al-Kateb, F., D. Dyab, B. Almadani, N. Al-Enezi. 2024. Spondyloenchondrodysplasia With Immune Dysregulation, but Without Skeletal Dysplasia, in a Six-Year-Old Boy: A Case Report. *Cureus*. 16(5):e60314. [https://doi.org/10.7759/cureus.60314](https://doi.org/10.7759/cureus.60314)

24.	Dri, J., E. Dos Santos, M. Pereyra, M.J. Guillamondegui, C. Ballester, A. Tolin, C. Gatica. 2024. Spondyloenchondrodysplasia with immune dysregulation related to ACP5. A report of 4 cases. *Arch Argent Pediatr*. 122(1):e202303031. [https://doi.org/10.5546/aap.2023-03031.eng](https://doi.org/10.5546/aap.2023-03031.eng)

25.	Romano, M., A. Geerlink, E. Demirkaya, R.A. Berard, F. Garcia-Bournissen. 2024. Pharmacokinetics of Baricitinib in Cerebrospinal Fluid and Plasma in a Patient with SPENCD. *J Clin Immunol*. 44(3):78. [https://doi.org/10.1007/s10875-024-01680-2](https://doi.org/10.1007/s10875-024-01680-2)

26.	Firat Senturk, E., B.B. Ayvaz, S. Firtina, S. Ugurlu. 2025. A rare case of late-onset spondyloenchondrodysplasia with immune dysregulation presenting as adult-onset monogenic lupus. *Lupus*. 34(1):108-14. [https://doi.org/10.1177/09612033241301506](https://doi.org/10.1177/09612033241301506)

27.	Pekpak Şahinoğlu, E., A.C. Oren, B. Şahinoğlu, Ö. Keskin, Ç. Damar, S. Akbayram. 2025. Clinical, laboratory, and molecular characteristics of patients with spondyloenchondrodysplasia: a case series study. *Eur J Pediatr*. 184(2):152. [https://doi.org/10.1007/s00431-025-05982-4](https://doi.org/10.1007/s00431-025-05982-4)

28.	Bilginer, Y., A. Düzova, R. Topaloğlu, E.D. Batu, K. Boduroğlu, Ş. Güçer, I. Bodur, Y. Alanay. 2016. Three cases of spondyloenchondrodysplasia (SPENCD) with systemic lupus erythematosus: a case series and review of the literature. *Lupus*. 25(7):760-5. [https://doi.org/10.1177/0961203316629000](https://doi.org/10.1177/0961203316629000)

29.	Kaya Akca, U., P.O. Simsek Kiper, G. Urel Demir, E. Sag, E. Atalay, G.E. Utine, M. Alikasifoglu, K. Boduroglu, Y. Bilginer, S. Ozen. 2021. Genetic disorders with symptoms mimicking rheumatologic diseases: A single-center retrospective study. *Eur J Med Genet*. 64(4):104185. [https://doi.org/10.1016/j.ejmg.2021.104185](https://doi.org/10.1016/j.ejmg.2021.104185)

30.	Shimizu, M., N. Inoue, M. Mizuta, H. Irabu, M. Okajima, Y. Honda, H. Nihira, K. Izawa, A. Yachie, T. Wada. 2021. Successful treatment of spondyloenchondrodysplasia with baricitinib. *Rheumatology (Oxford)*. 60(2):e44-e6. [https://doi.org/10.1093/rheumatology/keaa356](https://doi.org/10.1093/rheumatology/keaa356)

31.	Wang, W., W. Wang, L.P. Zou, T.Y. He, M.S. Ma, W.D. Li, Z.X. Yu, J. Yang, H.M. Song. 2021. [Clinical characteristics of 25 patients with type Ⅰ interferonopathies]. *Zhonghua Er Ke Za Zhi*. 59(12):1043-7. [https://doi.org/10.3760/cma.j.cn112140-20211004-00843](https://doi.org/10.3760/cma.j.cn112140-20211004-00843)

32.	Hong, S.W., K.H. Huh, J.K. Lee, J.H. Kang. 2018. Craniofacial anomalies associated with spondyloenchondrodysplasia: Two case reports. *Medicine (Baltimore)*. 97(50):e13644. [https://doi.org/10.1097/MD.0000000000013644](https://doi.org/10.1097/MD.0000000000013644)
